# MXene Key Composites: A New Arena for Gas Sensors

**DOI:** 10.1007/s40820-024-01430-4

**Published:** 2024-06-06

**Authors:** Yitong Wang, Yuhua Wang, Min Jian, Qinting Jiang, Xifei Li

**Affiliations:** 1 Hubei Province Key Laboratory of Systems Science in Metallurgical Process, Wuhan University of Science and Technology, Wuhan, 430081 People’s Republic of China; 2 Key Materials and Components of Electrical Vehicles for Overseas Expertise Introduction Center for Discipline Innovation, Institute of Advanced Electrochemical Energy and School of Materials Science and Engineering, Xi’an University of Technology, Xi’an, 710048 People’s Republic of China; 3 College of Materials Science and Engineering, Fuzhou University, Fuzhou, 350108 Fujian People’s Republic of China

**Keywords:** MXene, Compound material, Gas sensor, Gas sensitive preparation, Gas sensitivity performance

## Abstract

With the development of science and technology, the scale of industrial production continues to grow, and the types and quantities of gas raw materials used in industrial production and produced during the production process are also constantly increasing. These gases include flammable and explosive gases, and even contain toxic gases. Therefore, it is very important and necessary for gas sensors to detect and monitor these gases quickly and accurately. In recent years, a new two-dimensional material called MXene has attracted widespread attention in various applications. Their abundant surface functional groups and sites, excellent current conductivity, tunable surface chemistry, and outstanding stability make them promising for gas sensor applications. Since the birth of MXene materials, researchers have utilized the efficient and convenient solution etching preparation, high flexibility, and easily functionalize MXene with other materials to prepare composites for gas sensing. This has opened a new chapter in high-performance gas sensing materials and provided a new approach for advanced sensor research. However, previous reviews on MXene-based composite materials in gas sensing only focused on the performance of gas sensing, without systematically explaining the gas sensing mechanisms generated by different gases, as well as summarizing and predicting the advantages and disadvantages of MXene-based composite materials. This article reviews the latest progress in the application of MXene-based composite materials in gas sensing. Firstly, a brief summary was given of the commonly used methods for preparing gas sensing device structures, followed by an introduction to the key attributes of MXene related to gas sensing performance. This article focuses on the performance of MXene-based composite materials used for gas sensing, such as MXene/graphene, MXene/Metal oxide, MXene/Transition metal sulfides (TMDs), MXene/Metal–organic framework (MOF), MXene/Polymer. It summarizes the advantages and disadvantages of MXene composite materials with different composites and discusses the possible gas sensing mechanisms of MXene-based composite materials for different gases. Finally, future directions and inroads of MXenes-based composites in gas sensing are presented and discussed.

## Highlights

- With its layered structure, abundant functional groups, and excellent electrical conductivity, MXene is of great research interest in the field of gas sensing.
- The preparation technology of gas sensors is constantly being optimized, opening up avenues for the development of gas sensing.
- MXene-based composite materials (MXene/graphene, MXene/metal oxides, MXene/MOF, and MXene/polymer) are applied in various gas sensors.

## Graphical abstract

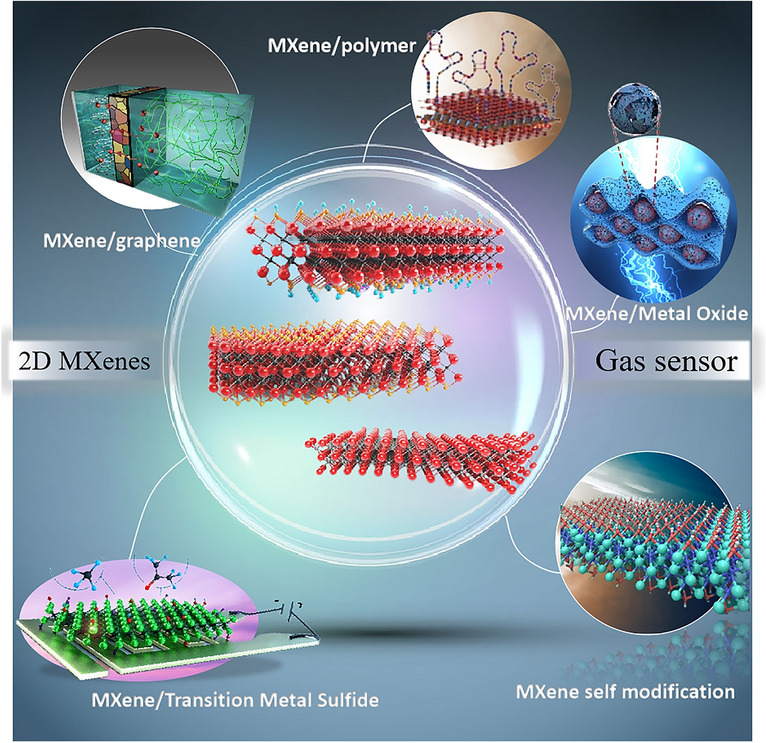

## Introduction

As a key component in information acquisition and signal conversion, sensors play an irreplaceable role in Internet of Things technology [1–4]. Among them, gas sensing can convert gas molecular signals over optical signals, electrical signals, etc., widely applicable to aerospace, industrial production, agricultural planting, and human health monitoring, to realize monitoring, forecasting, and automatic control of toxic and harmful gases, as well as prediction of human respiratory system diseases [5]. Sensors are mainly classified into electrical (resistive/capacitive), electrochemical, mass-sensitive, and optical types [6]. Electrical gas sensors are widely studied due to their simple structure and easy processing of output signals. Gas sensing materials in electrical sensor components can adsorb gas molecules through physical/chemical interactions and undergo charge transfer, thereby causing changes in the electrical signal of the device. Currently, electrical gas-sensitive materials include metal oxide semiconductors (MOSs), precious metals, carbon materials, organic materials, and two-dimensional materials. Since the discovery of graphene, two-dimensional materials, such as transition metal chalcogenides (TMDs) [7], boron nitride (BN) [8], layered double hydroxides (LDHs) [9], black phosphorus (BP) [10], and transition metal carbon/nitrides (MXenes) [11], have also been applied in the field of gas sensing. MXenes have become an emerging gas sensing material due to their unique layered structure, significant physical, optical, and electrical properties, as well as active surfaces [12, 13].

MXenes was proposed in 2011 by the Gogotsi group at Drexel University, USA [14]. Within the passed-century, MXenes and its composites have been receiving quite a lot of attraction in the field of energy storage and conversion [15, 16], electromagnetic shielding [17], and sensitive electronics [18]. Two-dimensional MXenes present a promising class on sensitive properties and a wide variety of structures, tunable structures, and controllable surface terminations [19]. In 2017, Lee et al. [20] found for the first time experimentally that Ti_3_C_2_ MXene has good gas-sensitive properties and exhibits gas-sensitive properties at room temperature [21–28]. Because common semiconductor gas-sensitive materials operate at high temperatures of 200–400 °C [29–32], MXene with room temperature gas-sensitive properties has the following advantages as a gas-sensitive material: (1) energy saving and simplification of the gas sensor structure [33, 34]; (2) painted on suitable matrix materials to develop portable and flexible gas sensors [35–41].

**Fig. 1:**
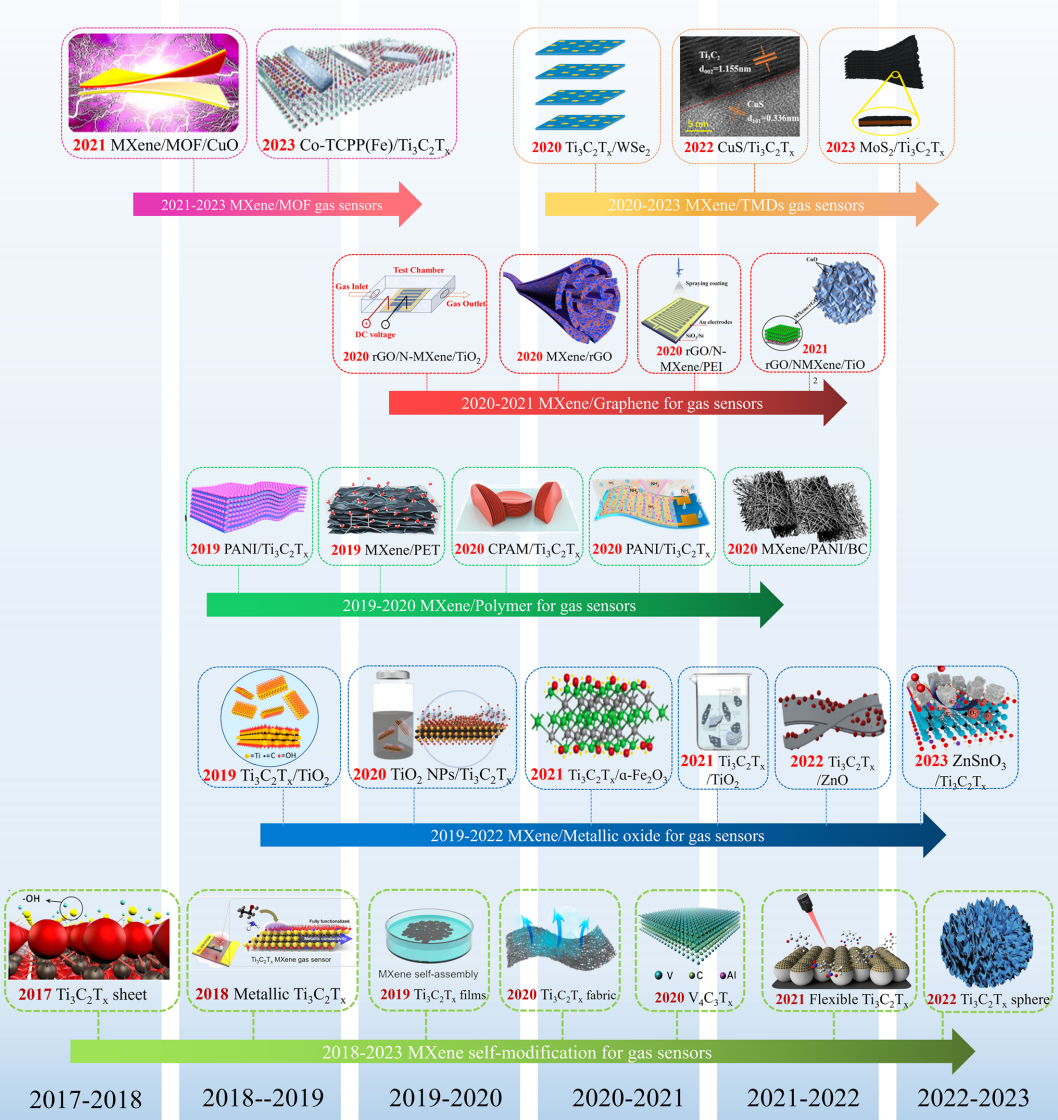
A significant research schedule of MXene compounded with other materials for gas sensors includes MXene self-modification, MXene/graphene, MXene/metal oxide, MXene/transition metal sulfide, MXene/MOF, MXene/polymer, etc. Reproduced with permission from Refs. [1, 35, 38, 42–44]

In recent years, the rapid development of MXenes has led to their rapid application in the field of gas sensing (Fig. 1) [1, 35, 38]. MXenes-based gas sensors are expected to achieve efficient and rapid detection of gases such as ammonia (NH_3_), nitrogen dioxide (NO_2_), and volatile organic compounds (VOCs) at room temperature [42–44]. However, due to its excellent electron transfer performance, two-dimensional layered structure, and abundant terminal groups, MXenes are not only sensitive to inorganic gases prone to electron loss or capture, but also highly sensitive to volatile organic compounds such as alcohols, ketones, and aldehydes [45]. This results in poor selectivity and specificity of MXenes in gas detection. Therefore, researchers often use surface modification, doping, and composite methods to enhance the gas sensing characteristics of MXenes [46–52]. Among them, compounding is an important strategy [53, 54]. The gas-sensitive composite phases of MXenes mainly include graphene and its derivatives, metal oxides, TMDs, MOFs, and polymers [55–57].

**Fig. 2:**
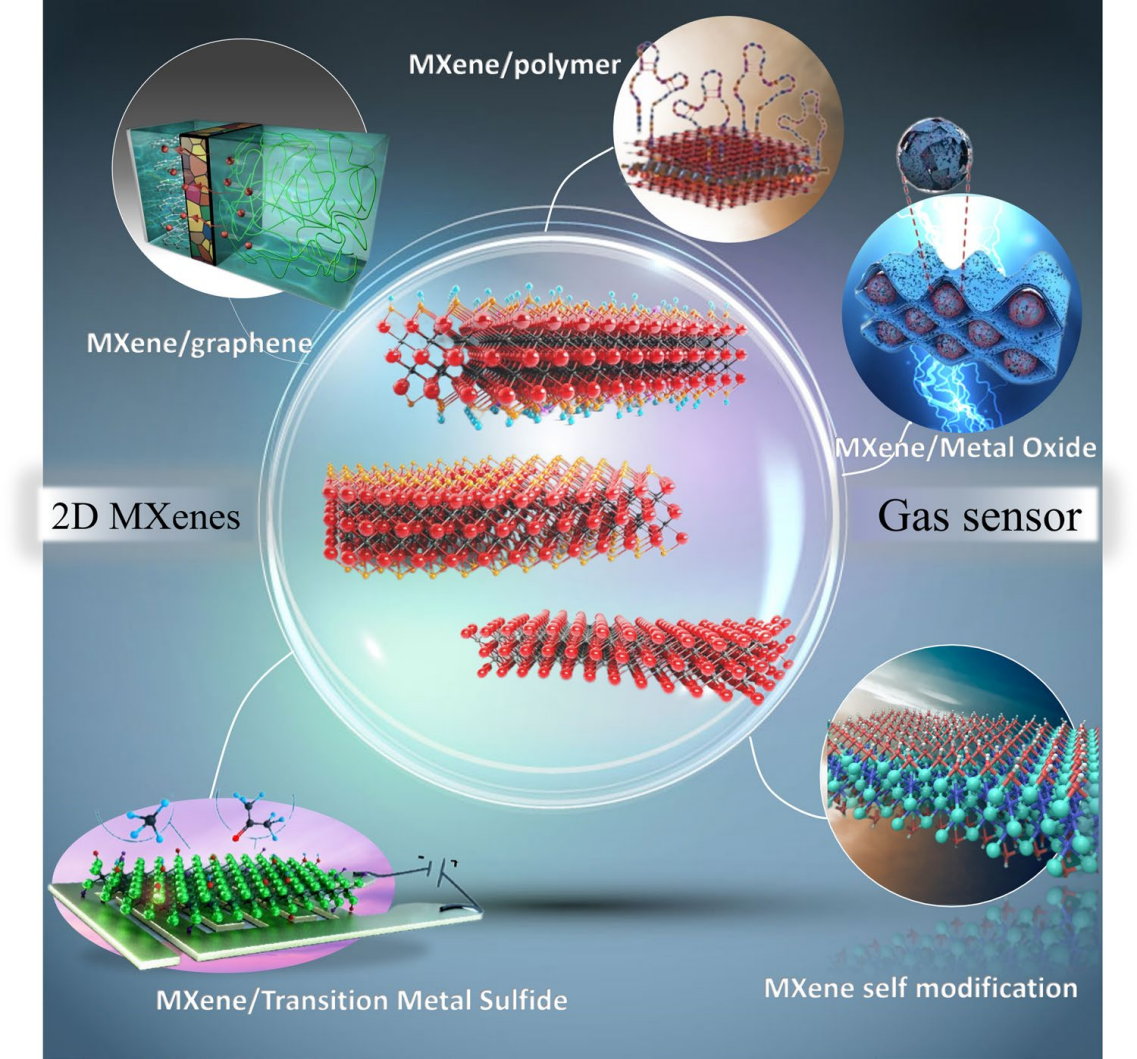
The structural diagram of MXene and the selection of high sensitivity MXene composite materials for gas sensing devices (MXene self-modification, MXene/graphene, MXene/metal oxide, MXene/TMDs, MXene/MOF, MXene/polymer). Reproduced with permission from Refs. [55–57]

The current paper reviews the recent research progress of MXenes-based composites for gas sensors. Figure 2 shows an overview of the review article, highlighting the preparation of gas sensors, with a focus on the synthesis, advanced performance, and gas sensing behavior of MXenes composite materials (MXene/graphene, MXene/metal oxides, MXene/transition metal sulfides (TMDs), MXene/metal–organic framework (MOF), MXene/polymer). Finally, the (potential) advantages and challenges related to the development of MXenes were systematically discussed.

## Preparation of Gas Sensors

Electrical gas sensors are sensors that convert gas composition and concentration into electrical signals [58–61]. In today's highly digitized and intelligent world, the increasingly deteriorating environmental and personal health issues have attracted widespread attention [27, 62–66]. In this regard, the development and design of gas sensors have received attention and favor from researchers [67, 68]. Gas sensors can detect various gases, such as gas composition detection in chemical production, coal mine gas concentration detection and alarm, environmental pollution monitoring, gas leakage, fire alarm, combustion detection, etc. With the continuous development of social technology, the types of substrates continue to increase. However, traditional methods for preparing gas sensing devices are not suitable for many substrates, and traditional methods require high preparation conditions, low production efficiency, and extremely high preparation costs [69–74]. Therefore, innovative manufacturing technologies for gas sensors are very important [75]. Appropriate manufacturing methods for gas sensors have provided strong support for the wide application of gas sensors by not only improving the performance of the sensors, effectively simplifying the process steps and reducing the cost of production [76–78].

**Fig. 3:**
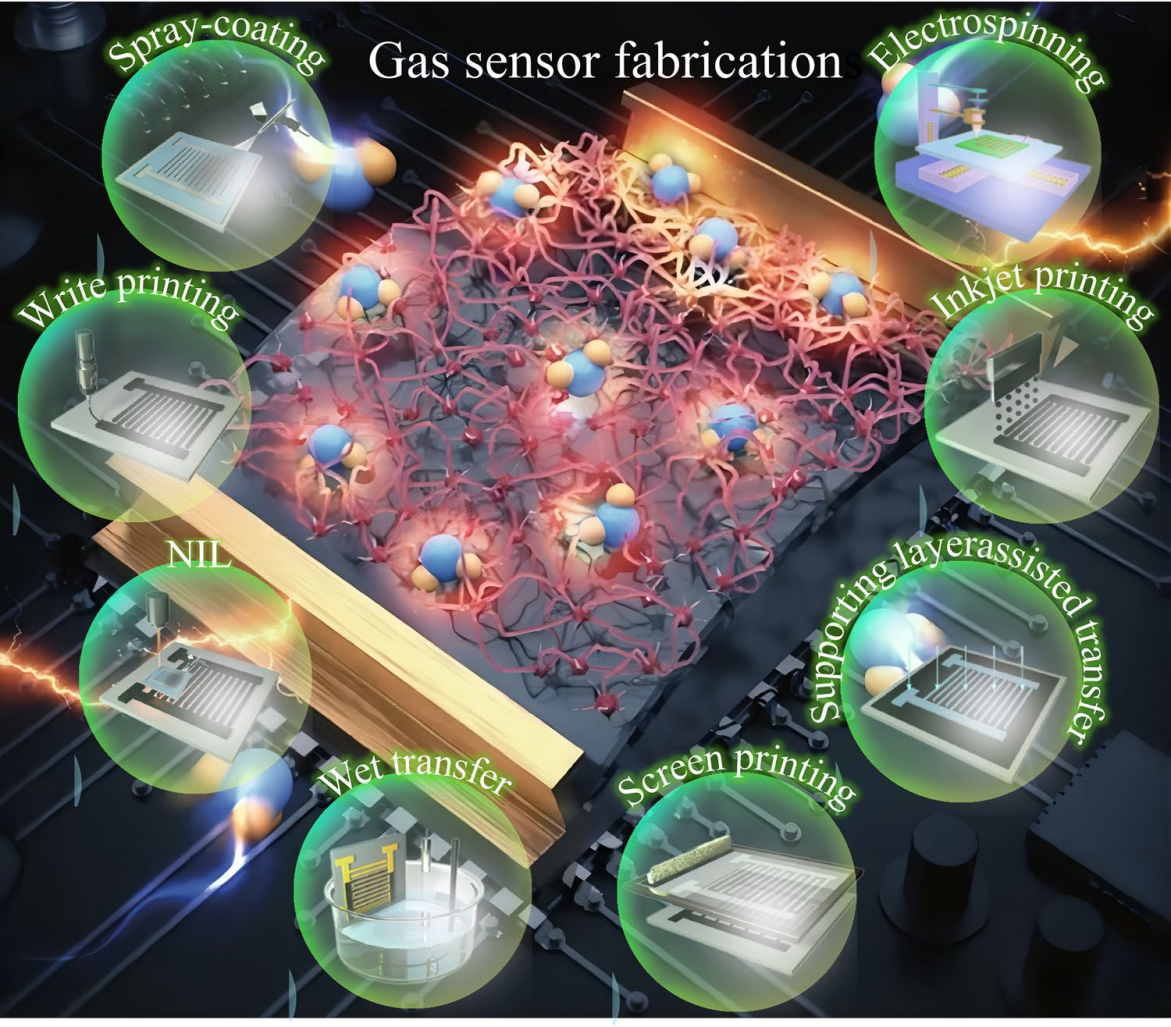
Preparation methods for gas sensors: application of coating technology (trickle coating, spinner coating, sprays, soap coating); imprinting technology (inkjet printing, silk screen printing, writing printing, nano-imprinting (NL)); transfer technology (electrospinning, other spinning); assignment technology (drying transfer, humid transfer, support layer-assisted transfer)

At present, the existing technologies for preparing sensors include: coating technology [77–82], printing technology [83–87], rotating technology [88–93], transfer technology [94–96] (Fig. 3). These technologies have led to enormous efforts in manufacturing optimization, resulting in impressive advances in gas sensors [97–113]. Table 1 summarizes the advantages and limitations of these specific technologies [114].

**Table 1:** Summarized the technology for preparing gas sensing equipment, summarized its advantages and disadvantages, as well as the demand for materials

Technique	Classification	Advantage	Limitation	Requirement for sensitive material
Coating techniques	Trickle-coating	Facile	Geometry-uncontrolled	Soluble or in solvent suspension
		High-efficient		
	Spinner-coating	Facile	Sensing material-waste	Soluble or in solvent suspension
		Thickness uniformity		
	Sprays	Geometry-controlled	Nozzle-blockage	Soluble or in solvent suspension
			Mask-relied	
	Soap-coating	Facile	Geometry-uncontrolled	Soluble or in solvent suspension
		Versatile		
Imprinting techniques	Inkjet printing	Digital controllable backup	Nozzle-blockage	Soluble or in solvent suspension
	Screen printing	High-efficient	Mesh-relied	Soluble or in solvent suspension
		Digital controllable backup		
	Write printing	Facile	Low-efficient	No special
	Nanoimprinting (NIL)	High resolution	Complicated	Soft
Transfer techniques	Electrospinning	High degree of efficiency	Irregular shape	Soluble or in solvent suspension
		Low cost	Blockage of nozzle	
	Other spinning yarn	High degree of efficiency	Geometry-irregular	Soluble or in solvent suspension
		Low cost	Nozzle-blockage	
Assignment technology	Drying transfer	Facile	Geometry-fragmentary	No special requirement
	Humid transfer	Facile	Low-efficient	Low density
			Location-uncontrolled	
	Supporting layer-assisted transfer	Versatile	Complicated	No special requirement

Coating technology is a simple and efficient way to prepare sensitive soluble materials into thin-film structures at the surface of a substrate [115, 116]. The preparation process of this technique is not demanding in terms of equipment and fabrication conditions, making it suitable for many substrates, including many flexible substrates, especially if the boundary range of the sensitive material film is not strictly required [76, 117]. Therefore, the coating technique is also one of the most prevalent methods for the fabrication of gas sensors today. Specific methods of coating technology include trickle coating, spin coating, spray coating, and dip coating. Trickle-coating method is one of the simplest ways to prepare gas sensors by selecting a soluble sensitive material and applying the material solution dropwise onto the substrate via a pipette, which is simple to operate; spin-coating is an alternative and convenient method of making sensitive films, in which a soluble material is dripped onto a rotating substrate, which is then dried so that the substrate vaporizes the solvent and a sample of the substrate containing a thin film of the sensitive material is obtained; spray-coating method can be assisted by ultrasound and combines the hydrophilic and hydrophobic properties of the materials adhered to prepare a homogeneous and sensitive film, and it is a cost-effective method [79, 80, 118]. However, its shortcoming is that for films with specific needs (e.g., specific requirements for shape and location), a concealment procedure had to be applied to the areas that did the coating not need to be applied; dip coating is a versatile and cost-effective method of preparing gas-sensitive sensors, which proceeds by dipping the substrate into a solution of sensitive material, then adjusting the speed to lift the substrate out of suspension, and finally drying to eliminate any residual solvent on the substrate surface.

Printing technology is an innovative and modern manufacturing technology that enables the preparation of functional material suspensions based on substrates using the appropriate printers and finds its application in a wide range of electronic manufacturing applications [81, 119, 120]. Pre-designed gas sensors, such as specific patterns, film thicknesses, and boundary ranges, can be prepared on a massive scale by printing technology. Printing technologies can be categorized into four main types, which are inkjet printing, screen printing, writing printing, and nano-imprinting. Inkjet printing is an intriguing digital method for contactless spraying of ink and functional materials onto a variety of substrates through micron-sized nozzles; screen printing is recognized as a highly attractive and competitive manufacturing technology compared to inkjet printing for the rapid mass fabrication of microelectronic devices due to its pre-designed grid pattern and ease of manufacturing process; writing printing on a substrate is a familiar and practical printing technique in which a combination of a functional material solution is deposited on the substrate to form a structure by combining it with a pen or any other writing instrument; nanoimprint lithography (NIL) is a lithographic technique that offers the advantages of high productivity, low cost, and simplicity of the process to fabricate nanostructures in high volume, high resolution (< 5 nm), and lower cost. Simply put, NIL technology uses high-resolution electron beams and other methods to pattern complex nanostructures on a stamp, and then deforms the sensitive material with the patterned stamp to form the patterned material. Unlike traditional photolithography (where the direction or energy of the ions of the sensitive material is altered by photons or electrons to achieve pattern production), NIL technology mechanically deforms the sensitive material through direct contact, thus avoiding the resolution limitations of traditional techniques such as light diffraction or beam scattering.

Spinning is the process of extracting a precursor functional solution (e.g., polymer solution or melt) from a nozzle and depositing it on a collector to create long, continuous, one-dimensional fibers with micron/nanometer diameters [121–123]. Textiles can be coated with sensitive materials to form gas sensors. In addition, electronic devices based on sensitive optical fibers can be directly fabricated for gas detection via incorporating gas-sensitive materials into the precursor solution, which is a straightforward and effective method. Of the various spinning technologies, the electrospinning technology is of great interest and is widely utilized for the preparation of a wearable device.

Many substrates are incompatible given that in particular conventional fabrication techniques (e.g., chemical vapor deposition (CVD)), certain substrates cannot withstand drastic fabrication conditions (e.g., high temperatures, chemical etching reagents). The optimum way to resolve these incompatibilities lies in the transfer of nanostructures or thin films on rigid/donor substrates (e.g., silicon, glass) prepared by conventional fabrication techniques to acceptor substrates (e.g., PET, PMDS), which is defined as a transfer technique [124, 125]. Effective transfer techniques are critical to the fabrication of flexible gas sensors, which will enable many traditional fabrication processes that are only applicable to hard substrates to be used in the manufacture of wearable/flexible sensors [114]. Transfer techniques consist of dry transfers, wet transfers, and support coatings-assisted transfers. Dry transfer utilizes the adhesion gap between the film layer and the underlying substrate to transfer the film from the primary substrate to the intended substrate; wet transfer is available for transferring a mono sensitive layer to a variety of substrates in service media; and support layer-assisted transfer is a prominent transfer technique that utilizes an elastomeric impression as a support layer to retrieve a material with micro/nanostructures back from the supplier substrate and attach it to a non-natural substrate [126].

However, most of the aforementioned widely practiced techniques (e.g., coating, printing, and spinning) rely on the sensing material being in the liquid phase, this restricts the amount of gas sensing materials available because some types of materials with excellent sensing capabilities are harder to realize in the bulk of the liquid phase. Spin-coating and screen-printing methods result in ink waste due to the use of solution-phase materials, while inkjet printing and electrospinning processes both require the use of nozzle devices, with the risk of nozzle clogging. Moreover, technology of transfer, particularly transfer with the assistance of a supporting layer where at least two etching cycles are involved, is partly complex and time-consuming. Hence, a long way lies ahead in commercializing the product for the exploitation of gas sensors with enhanced performance and large-scale production.

## Structure and Properties of MXene

### Structure of MXene

**Fig. 4:**
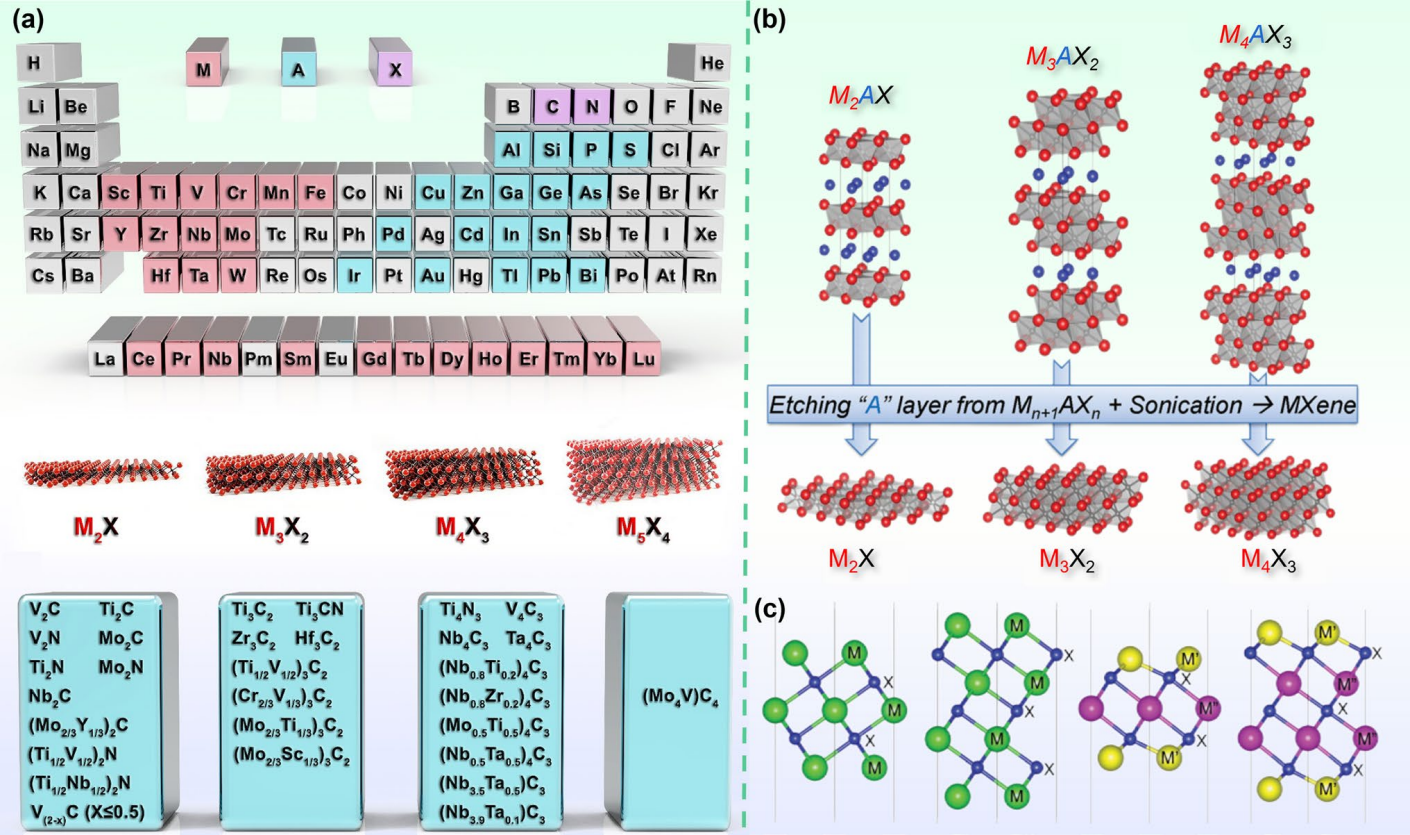
**a** Explain the "M," "A," and "X" elements of MAX phase through the periodic table, as well as the schematic diagram of MXenes structure and the currently reported MXenes. Reproduced with permission from Ref. [127]. b Crystal structure of MXene generated from MAX phase. c Side views of pristine M_3_X_2_, M_4_X_3_, M’_2_M”X_2_, and M’_2_M”_2_X MXenes, where M, M’, and M” denote transition metals, and X represents C or N. Reproduced with permission from Ref. [128]

MXene material is a type of metal carbide or metal nitride material with a two-dimensional layered structure. It is a two-dimensional transition metal group carbon/nitride obtained by selectively etching the A atomic layer in the ternary conductive ceramic MAX phase. The phase structure of MXene is shown in Fig. 4a, and the general formula of MXene structure is M_n+1_X_n_T_x_, where M is a transition metal (such as Ti, V, and Mo), X represents C or N, n = 1, 2, or 3, T_x_ represents surface terminal groups (-OH, = O, and/or -F) [127]. Due to the hexagonal crystal structure formed by the interlacing of the M layer and X layer with the A layer in the precursor MAX phase of MXenes, the MXene phase also has a similarly symmetrical hexagonal lattice (Fig. 4b). The M atoms in MXenes are arranged in a tight structure, while the X atoms fill the gap positions of the octahedron. There are three arrangements in the MXenes structure: B_γ_A-A_γ_B (M_2_X-M_2_X), B_γ_A_β_C-C_β_A_γ_B (M_3_X_2_-M_3_X_2_) and B_α_C_β_A_γ_B-B_γ_A_β_C_α_B (M_4_X_3_-M_4_X_3_) [128]. As shown in Fig. 4c, the two-dimensional MXene consists of a thin sheet that has hexagonal cells, with an X layer sandwiched between two M transmission metal layers.

### MXene Characteristics for Gas Sensing

In the aqueous environment of chemical etching solutions, the outer surface of the detached MX layer is usually functionalized by -F, -OH or = O functional groups. These surface rich functional groups (-F, -OH or = O) can become attachment sites for the direct growth of other nanostructured materials or functional molecules [129, 130], which can be modified to provide feasibility for improving the selectivity of gas sensors. In addition, this surface functionalization has a significant impact on the electronic and ion transport properties of MXenes, namely, the conductivity of MXenes is directly related to the electron transfer process that occurs on their surface [131]. MXenes have certain metal properties and narrow bandgap semiconductor properties, which give them the inherent advantage of good conductivity. For example, Ti_3_C_2_T_x_ has a room temperature conductivity of up to 10,000 S cm^−1^ [132, 133].

Many theoretical calculations have shown that the ideal MXene is located near the Fermi level, with a considerable electron density and a cash property [128, 130]. Lane et al. calculated the ideal single-layer defect free MXene nanosheets using density functional theory, and the results showed that MXene exhibits metal conductivity, with Fermi levels higher than its precursor MAX phase [131]. However, when its surface is functionalized, some MXenes exhibit semiconductor properties. In addition, due to the different number of electrons received by different surface groups (-F, -OH, or = O) in equilibrium states, different surface groups have different effects on the electronic properties of MXene, and the orientation of the end groups also affects the electronic properties of MXene [132]. Table 2 lists the bandgap widths of some MXenes. MXenes with different bandgap widths can be used to prepare gas sensing arrays, achieving specific recognition of industrial raw gas, exhaust gas, and human exhaled gas. In summary, using MXenes as a gas sensing material has certain inherent advantages.

**Table 2:** Bandgap width of some MXenes

MXenes	Functional group	Bandgap (eV)	References
Ti_2_C	–F	0.72	[134]
	–OH	1.07	[134]
	=O	0.24	[135]
Ti_3_C_2_	–F	0.39	[134]
	–OH	1.35	[134]
Nb_2_C	–F	0.96	[134]
	–OH	1.29	[134]
V_2_C	–F	0.24	[134]
	–OH	1.09	[134]
Cr_2_C	–F	3.49	[136]
	–OH	1.43	[136]
Cr_2_TiC_2_	–F	1.35	[137]
	–OH	0.84	[137]
Sc_2_TiC_2_	–F	1.03	[135]
	–OH	0.45	[135]
	=O	1.8	[135]
Hf_2_C	=O	1.0	[135]
Hf_3_C_2_	=O	0.16	[138]
Zr_2_C	=O	0.88	[135]

## MXenes Composite in Gas Sensing Applications

In recent years, MXene composite materials containing graphene, semiconductor metal oxides, transition metal sulfides, organic metal frameworks, polymers, and other materials have received increasing research in gas sensing applications. Due to the more metallized nature and narrow band gap of MXenes, the addition of metal oxides, graphene derivatives, and chalcogenides provides more activated adsorption sites, defects, and modulation of working functions, thereby improving gas sensing performance. Table 3 summarizes the performance of gas/VOC/humidity sensors for MXene-based composites.

**Table 3:** Gas/VOC/humidity sensor based on MXene-composite

Classification	MXene-based composite	Target gas	Test range	Carrier gas	Sensitivity	Response time (t_Res._)	Recovery time (t_Rec._)	Operating temperature	References
MXene/rGO	Ti_3_C_2_T_x_/rGO fibers	NH_3_	10–500 ppm	Dry air	6.77% (50 ppm)	> 10 min	> 25 min	RT	[139]
	Ti_3_C_2_T_x_/rGO/CuO aerogel	Acetone (CH_3_COCH_3_)	10–500 ppm	Dry air	52.09% (100 ppm)	6.5 s	7.5 s	RT	[140]
	rGO/N-MXene/TiO_2_ film	Formaldehyde (HCHO)	4–40 ppm	Wet air	26% (4 ppm)	27.6 s	4.8 s	20 °C	[141]
	rGO/N-MXene/PEI film	CO_2_	8–3000 ppm	Wet air	1.3% (8 ppm)	8.8 min	9 min	20 °C	[142]
	Ti_3_C_2_T_x_/TiO_2_-spaced rGO	NO_2_	0.05–20 ppm	Wet air	165% (1 ppm)	180 s	260 s	RT	[143]
MXene/metallic oxide	TiO_2_/Ti_3_C_2_T_x_ bilayer film	NH_3_	0.5–10 ppm	Wet air	3.1% (10 ppm)	33 s	277 s	25 °C	[144]
	CuO/Ti_3_C_2_T_x_ MXene hybrids	Toluene (C_6_H_5_CH_3_)	10–50 ppm	Wet air	11.4% (50 ppm)	270 s	10 s	250 °C	[145]
	Co_3_O_4_@PEI/Ti_3_C_2_T_x_	NO_x_	0.03–100 ppm	Wet air	27.9% (100 ppm)	< 2 s	73 s	RT	[146]
	MXene sphere/ZnO	NO_2_	5–100 ppm	Wet air	41% (100 ppm)	34 s	103 s	25 °C	[53]
	MXene/Co_3_O_4_	HCHO	0.01–10 ppm	Wet air	9.2% (10 ppm)	83 s	5 s	25°C	[147]
	W_18_O_49_/Ti_3_C_2_T_x_	CH_3_COCH_3_	0.17–20 ppm	Wet air	11.6% (20 ppm)	5.6 s	6 s	200–400 °C	[45]
	partially oxidized Ti_3_C_2_T_x_	Organic analytes	2 ppm	Dry air	40% (ethanol), 110% (methanol, 100% (isopropyl alcohol), and 180% (acetone)	–	–	20–350 °C	[148]
	Ti_3_C_2_T_x_//TiO_2_	Ethanol (C_2_H_5_OH)	10–800 ppm	Dry air	22.47% (100 ppm)	–	–	RT	[149]
	TiO_2_/Ti_2_CT_x_	NH_3_	1–100 ppm	Dry air	~ 0.4% (0.1 ppm)	–	–	RT	[150]
	Ti_3_C_2_T_x_/WO_3_	NH_3_	1–5 ppm	Wet air	22.3% (1 ppm)	119 s	228 s	RT	[151]
	MXene/SnO_2_	NH_3_	0.5–100 ppm	Wet air	40% (50 ppm)	36 s	44 s	RT	[152]
	TiO_2_/Ti_3_C_2_T_x_	NO_2_	0.125–5 ppm	Dry air	~ 37% (5 ppm)	–	–	RT	[44]
	SnO-SnO_2_/Ti_3_C_2_T_x_	CH_3_COCH_3_	10–100 ppm	Dry air	12.1 (100 ppm)	18 s	9 s	RT	[153]
	Ti_3_C_2_T_x_-TiO_2_	Hexanal	10–40 ppm	Dry air	~ 3.4% (10 ppm)	293 s	461 s	RT	[154]
	ɑ-Fe_2_O_3_/Ti_3_C_2_T_x_	CH_3_COCH_3_	5–200 ppm	Wet air	16.6% (5 ppm)	5 s	5 s	RT	[155]
	In_2_O_3_/Ti_3_C_2_T_x_	CH_3_OH	5–100 ppm	Wet air	29.6% (5 ppm)	6.5 s	3.5 s	RT	[156]
	ZnO/Ti_3_C_2_T_x_	NO_2_	5, 10 ppm	Wet air	54% (10 ppm)	–	–	RT	[157]
	ZnSnO_3_/MXene	HCHO	5–100 ppm	Wet air	62.4% (5 ppm)	6.2 s	5.1 s	RT	[158]
	Ti_3_C_2_/TiO_2_ nanowires	Humidity	7%–97% RH	Wet air	280 PF%RH	2 s	0.5 s	RT	[159]
	Ti_3_C_2_T_x_/K_2_Ti_4_O_9_	Humidity	11%–95%RH	Wet air	1.49% (95%RH)	65.2 s	84.8 s	RT	[160]
MXene/TMDs	MoS_2_/Ti_3_C_2_T_x_	NO_2_	10, 20 ppm	Wet air	40.1% (20 ppm)	525 s	155 s	RT	[161]
	Ti_3_C_2_T_x_/WSe_2_	C_2_H_5_OH	1–40 ppm	Wet air	~ 9% (40 ppm)	9.7 s	6.6 s	RT	[162]
MXene/MOF	Ti_3_C_2_T_x_/Cu MOF	NH_3_	1–100 ppm	Dry air	24.8% (100 ppm)	45 s	29 s	RT	[163]
	Co-TCPP(Fe)/Ti_3_C_2_T_x_	NO_2_	10 ppm	Dry air	2.0% (10 ppm)	95 s	15 s	RT	[164]
MXene/polymer	Polyanline/Ti_3_C_2_T_x_	NH_3_	0.5–50 ppm	Wet air	1.7% (10 ppm)	–	–	20 °C	[165]
	cationic polyacrylamide (CPAM)/Ti_3_C_2_T_x_	NH_3_	50–200 ppm	Dry air	4.7% (200 ppm)	12.7 s	14.6 s	RT	[166]
	PEDOT:PSS/MXene	NH_3_	10–1000 ppm	Dry air	36.6% (100 ppm)	116 s	40 s	27 °C	[103]
	MXene/polyaniline/bacterial (MXene/PANI/BC)	NH_3_	2.5–12.5 ppm	Dry air	56.63% (7.5 ppm)	–	–	RT	[101]
	PANI/Ti_3_C_2_T_x_	C_2_H_5_OH	50–200 ppm	Dry air	27.4% (150 ppm)	0.4 s	0.5 s	RT	[105]
	Ti_3_C_2_T_x_/PEDOT:PSS	CH_3_OH	180–500 ppm	Dry air	36.6% (100 ppm)	116 s	40 s	27 °C	[104]
	Ti_3_C_2_T_x_/polyurethane (PU)	CH_3_COCH_3_	0.05–50 ppm	Dry air	0.25% (50 ppb)	148 -190 s	164 -240 s	RT	[102]
	MXene/polyelectrolyte	Humidity	20%–70% RH	Wet air	39.5%	110 ms	220 ms	RT	[100]
	poly(vinyl alcohol)/Ti_3_C_2_T_x_(PVA/MXene)	Humidity	11%–97% RH	Wet air	40% (90% RH)	0.9 s	6.3 s	RT	[99]
	Ti_3_C_2_T_x_/chitosan (CS)	Humidity	14%–73% RH	Wet air	~ 0.16% (73% RH)	–	–	RT	[113]
	Ti_3_C_2_T_x_/chitosan (MCQMS)	Humidity	1%–98% RH	Wet air	317% (90% RH)	0.75 s	1.6 s	RT	[112]
Others	Ti_3_C_2_/Ag	Humidity	35%–95% RH	Wet air	106,800%	80 ms	120 ms	RT	[111]
	Ti_3_C_2_T_x_/Ag NWs	Humidity	57% RH	Wet air	~ 3%	5 s	80 s	20°C	[108]
	Ti_3_C_2_T_x_-K/Mg	Humidity	0%–85% RH	Wet air	~ 8% RH	–	–	27°C	[110]
	TiOF_2_@Ti_3_C_2_T_x_	Humidity	11%–95% RH	Wet air	39.5%	16 s	20 s	RT	[98]
	Ti_3_C_2_T_x_@Pb CNC	H_2_	0.5%–40%	Dry air	23.0 ± 4.0% (4%)	(37 ± 7)s	(161 ± 23) s	RT	[167]
	Ni(OH)_2_/Ti_3_C_2_T_x_	NH_3_	1–80 ppm	Wet air	6.2% (10 ppm)	78 s	~ 500 s	RT	[106]
	Ti_3_C_2_T_x_/flfluoroalkylsilane (FOTS)	C_2_H_5_OH	5–120 ppm	Wet air	14% (120 ppm)	39 s	139 s	RT	[109]
	Fe_2_(MoO_4_)_3_@MXene	n-butanol	100 ppm	Wet air	43.1%	18 s	24 s	120 °C	[107]

### MXene/Graphene

**Fig. 5:**
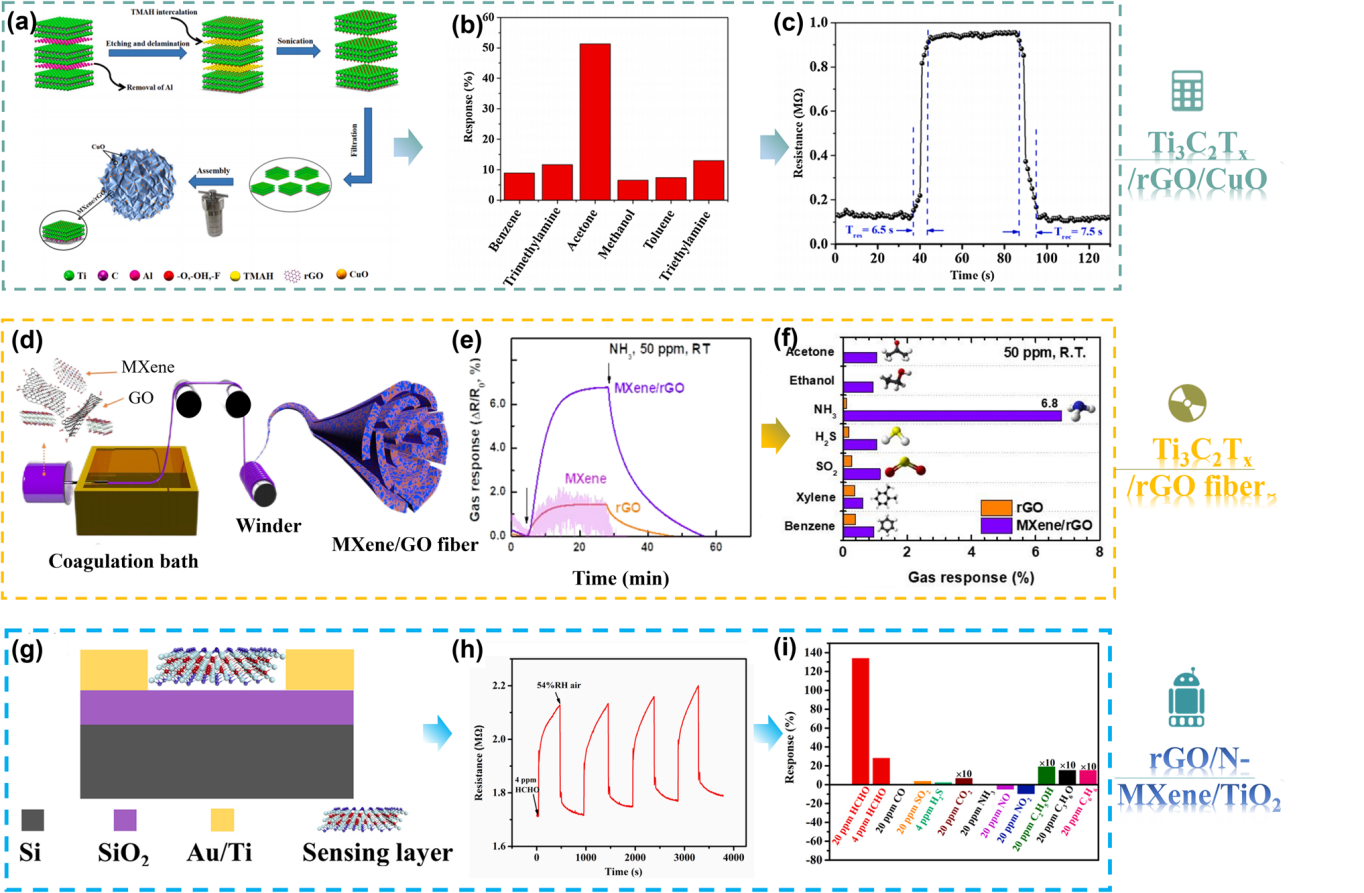
**a** Schematic illustration of fabrication process of 3D MXene/rGO/CuO aerogel. **b** The selectivity for 3D MXene/rGO/CuO aerogel-based sensor to different gases of 100 ppm at RT. **c** Resistance changes of 3D MXene/rGO/CuO aerogel when exposed to 100 ppm acetone at RT. Reproduced with permission from Ref. [140]. **d** Schematic illustration of the spinning process for MXene/GO hybrid fiber. **e** Comparison of the gas response of MXene film, rGO fiber, and MXene/rGO hybrid fiber (40 wt% MXene). **f** Gas selectivity comparison of rGO fiber and MXene/rGO hybrid fiber (40 wt% MXene) to various testing gases at concentrations of 50 ppm. Reproduced with permission from Ref. [139]. **g** Schematic images of IDEs sensor. **h** Sensing performance of the ternary sensor toward HCHO vapor under 54%RH at 20 °C. **i** Selectivity investigation among a series of interference gases under 54%RH at 20 °C. Reproduced with permission from Ref. [141]

Graphene is widely used in various fields because of its excellent thermal conductivity, high specific surface area, and easily modified structure [139–143]. MXenes are an excellent sensing material with a very narrow bandgap, but when pure MXenes are used in gas sensing devices, critical potential barriers are generated during the gas reaction process, which hinders their further sensitive response. Subsequently, researchers found that combining the two can effectively overcome this problem. For example, Liu et al. prepared three-dimensional (3D) hybrid aerogel [140] (Fig. 5a) from MXene (Ti_3_C_2_T_x_), reduced graphite oxide (rGO) nanosheets, and ultrafine CuO nanoparticles. From the obtained 3D MXene/rGO/CuO aerogel, high pyruvic sensing performance was demonstrated at ambient temperature (Fig. 5b). Response of the sensor to 100 ppm of acetone was 52.09% (RT) (Fig. 5b), with a response time of ~ 6.5 s and a recovery time of ~ 7.5 s (Fig. 5c), demonstrating excellent reproducibility and selectivity. In 2020, Lee et al. [139] developed a Ti_3_C_2_T_x_ MXene/graphene hybrid fiber wearable gas sensor without a metal binder through a wet spinning process (Fig. 5d). The bandwidth capacity of the composite material has increased from 1.05 to 1.57 eV, while the fiber properties of the composite material enhance flexibility and response to NH_3_. A moderate response (6.8% at 50 ppm NH_3_) was displayed by the composites (Fig. 5e), with this being 7.9 and 4.7 times more responsive than that of pure MXenes and rGO, respectively (Fig. 5f).

Wang et al. [141] proposed an ionic conductive composite film, which is composed of reduced graphite oxide (rGO), nitrogen doped MXene Ti_3_C_2_T_x_ (N-MXene), and titanium oxide (TiO_2_) (Fig. 5g), and detects 4–40 ppm formaldehyde HCHO vapor at room temperature (20 °C) and humidity. In various humidity conditions toward 4 ppm HCHO, the ternary sensor achieved an average reversible response of 26% at 54% RH (Fig. 5h). In addition, it also shows good repeatability, long-term stability, and selectivity (Fig. 5i). The excellent gas sensing performance of rGO nanosheets can be attributed to three aspects: firstly, in humid environments, rGO nanosheets serve as a good conductive platform for transporting and collecting charge carriers; second, the layered N-MXene facilitates the co-sorption and spreading of HCHO and water moieties; third, the TiO_2_ nanoparticles provide abundant resorption sites, which promote decomposition of the sorbed water.

In the wet CO_2_ sensing process of composite materials, few rGO nanosheets serve as a good conductive platform for transferring and collecting load carrier. The layered N-MXene provides further reactive sites to co-adsorb carbon dioxide and water, thus facilitating reactions involving water. The abundant amino groups in PEI polymers facilitate the binding of CO_2_ molecules, leading to significant changes in charge carrier density through proton conduction behavior [98, 168–173]. However, MXene composite material sensors with graphene or graphene derivatives are mostly subjected to multi gas testing, with no targeted detection of a single gas, and there is little research on humidity sensing [128, 174–184].

### MXene/Metal Oxide

**Fig. 6:**
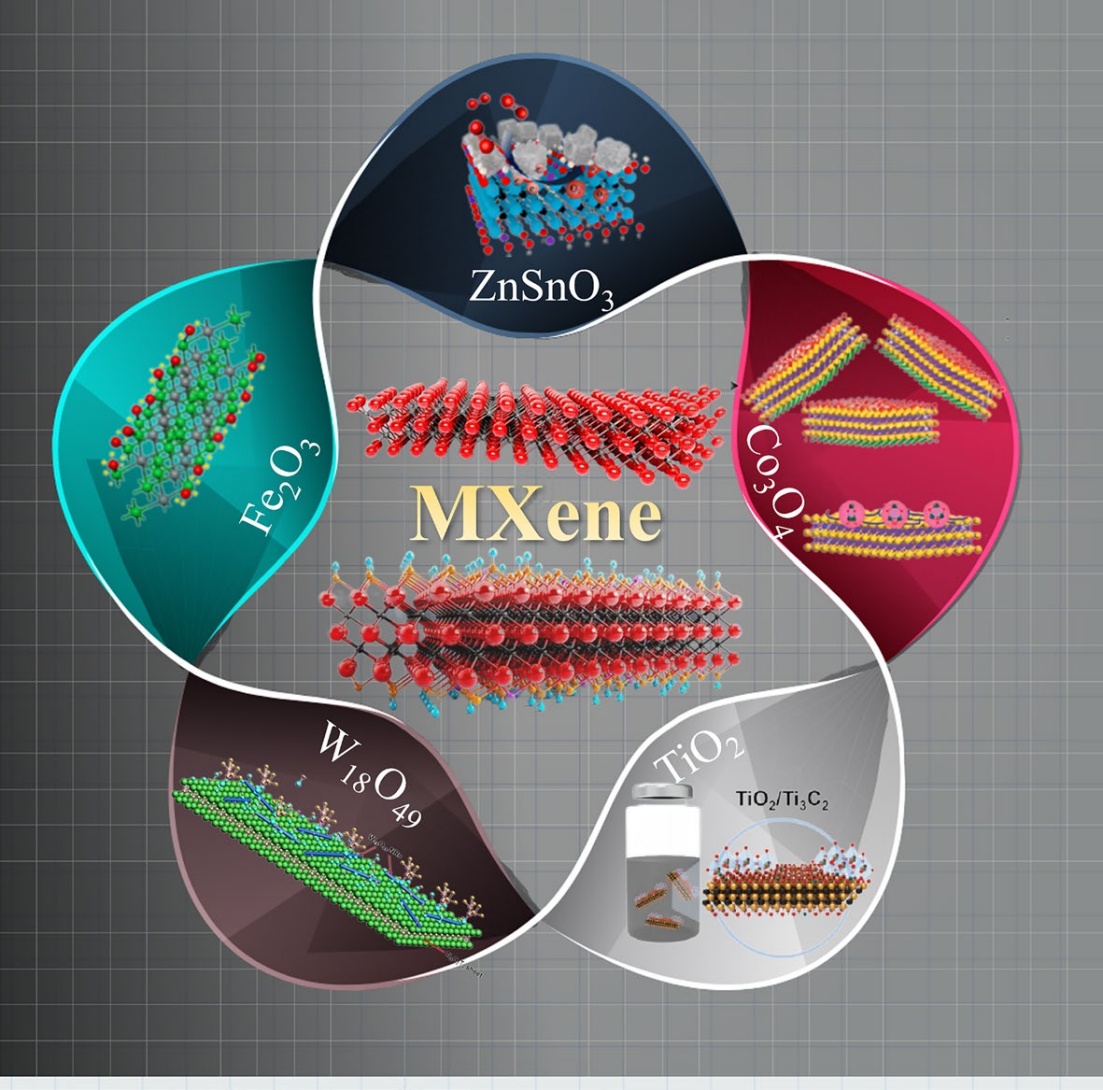
MXene/metal oxide for gas sensors. Reproduced with permission from Refs. [44, 146, 155, 158, 187]

Metal oxides represent the oldest and most widely used sensing material and can be used in a variety of applications due to the high specific surface area, ease of fabrication, ease of functionalization, and extremely high sensitivity to a broad range of gases/volatile organic compounds. The sensing mechanism of metal oxides is mainly due to the changes in resistance caused by pre adsorbed oxygen species (oxygen molecules (O_2_), lattice oxygen < including surface lattice oxygen and bulk lattice oxygen > (O^2−^), atomic adsorption of oxygen (O^−^), molecular adsorption of oxygen (O_2_^−^)), and surface reactions of gas molecules [185]. Due to the high dependence of oxygen ionization on operating temperature, this mechanism typically requires metal oxide gas sensors to operate at relatively high temperatures, which is also the main drawback of metal oxide gas sensors [53, 145–147, 186]. However, research data suggests that the mixture of metal oxides with 2D MXenes has a more robust gas/volatile organic compound sensing response, and the emergence of this complex greatly overcomes the low selectivity and high operating temperature limitations of pure metal oxide sensing (Fig. 6).

**Fig. 7:**
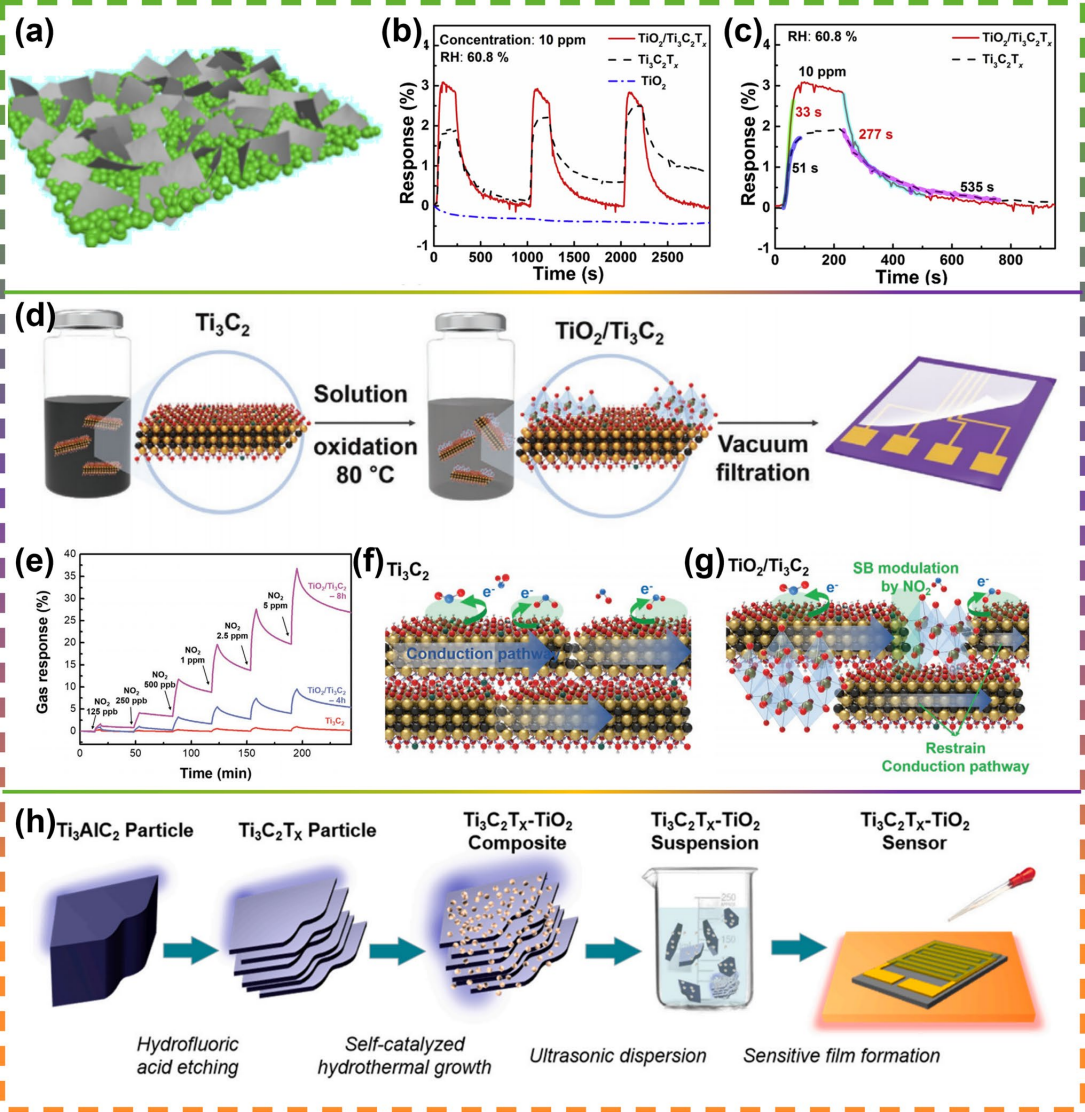
**a** Structure of TiO_2_/Ti_3_C_2_T_x_ Gas Sensor. **b** Normalized response recovery curves of the TiO_2_/Ti_3_C_2_T_x_, Ti_3_C_2_T_x_ and TiO_2_ gas sensors to 10 ppm NH_3_. **c** Response/recovery times of the Ti_3_C_2_T_x_ and TiO_2_/Ti_3_C_2_T_x_ gas sensors. Reproduced with permission from Ref. [144]. **d** A diagram of the composition process of TiO_2_/Ti_3_C_2_ MXene sensor. **e** Experimental real-time gas response curve of TiO_2_/Ti_3_C_2_ depending on NO_2_ concentration. **f** and **g** Suspension regime of NO_2_ gas by Ti_3_C_2_ and TiO_2_/TiO_2_/Ti_3_C_2_ thin films. Reproduced with permission from Ref. [44]. **h** Schematic diagram illustrating the process of the Ti_3_C_2_T_x_-TiO_2_ nanocomposites preparation and gas sensing device fabrication. Reproduced with permission from Ref. [154]

Titanium dioxide (TiO_2_) is an ideal material for gas sensor preparation due to its pollution-free properties, ability to generate photogenerated electrons when stimulated, and simple preparation process. However, TiO_2_-based gas sensors also have some drawbacks, such as poor sensing performance, long response time, and recovery time. In 2019, Tai [144] designed a gas sensing element based on a TiO_2_/Ti_3_C_2_T_x_ bilayer film (Fig. 7a). According to the results, when compared with the pure Ti_3_C_2_T_x_ sensor, this TiO_2_/Ti_3_C_2_T_X_ sensor exhibited a larger recognition value (1.63 times) with shorter response/recovery time (0.65/0.52 times) compared to the pure Ti_3_C_2_T_X_ sensor for 10 ppm NH_3_ at room temperature of 25 °C (60.8% relative humidity) (Fig. 7b, c). Choiet et al. [44] covered the amplification and inductive properties against NO_2_ by Ti_3_C_2_ through the modulation of the introduction of the Schottky barrier (SB) (Fig. 7d), which combines TiO_2_ into conducting MXenes to form a heterogeneous structure. The TiO_2_/Ti_3_C_2_ composite sensor shows a NO_2_ sensitivity 13.7 times higher than the original Ti_3_C_2_ MXene (Fig. 7e), while the response of the reducing gas is almost unchanged, the reason for this is the highest charge density of NO_2_ in other interfering VOCs due to the formation and movement of SB inside caused by the adsorption of NO_2_ molecules, together with other interfering VOCs, and as explained in the mechanisms of sensing (Fig. 7f, g). Kuang et al. [154] successfully prepared Ti_3_C_2_T_x_-TiO_2_ nanocomposites with regular morphology using Ti_3_C_2_T_x_ as the titanium source through a simple one-step hydrothermal synthesis method (Fig. 7h). Due to the formation of interface heterojunctions and modulation of carrier density, the detection response of Ti_3_C_2_T_x_-TiO_2_ sensors to various VOCs at room temperature is enhanced by about 1.5–12.6 times compared to pure MXene sensors. In addition, this nanocomposite sensor has a better response to hexanal (the Ti_3_C_2_T_x_-TiO_2_ sensor has a gas response of approximately 3.4% to 10 ppm hexanal).

**Fig. 8:**
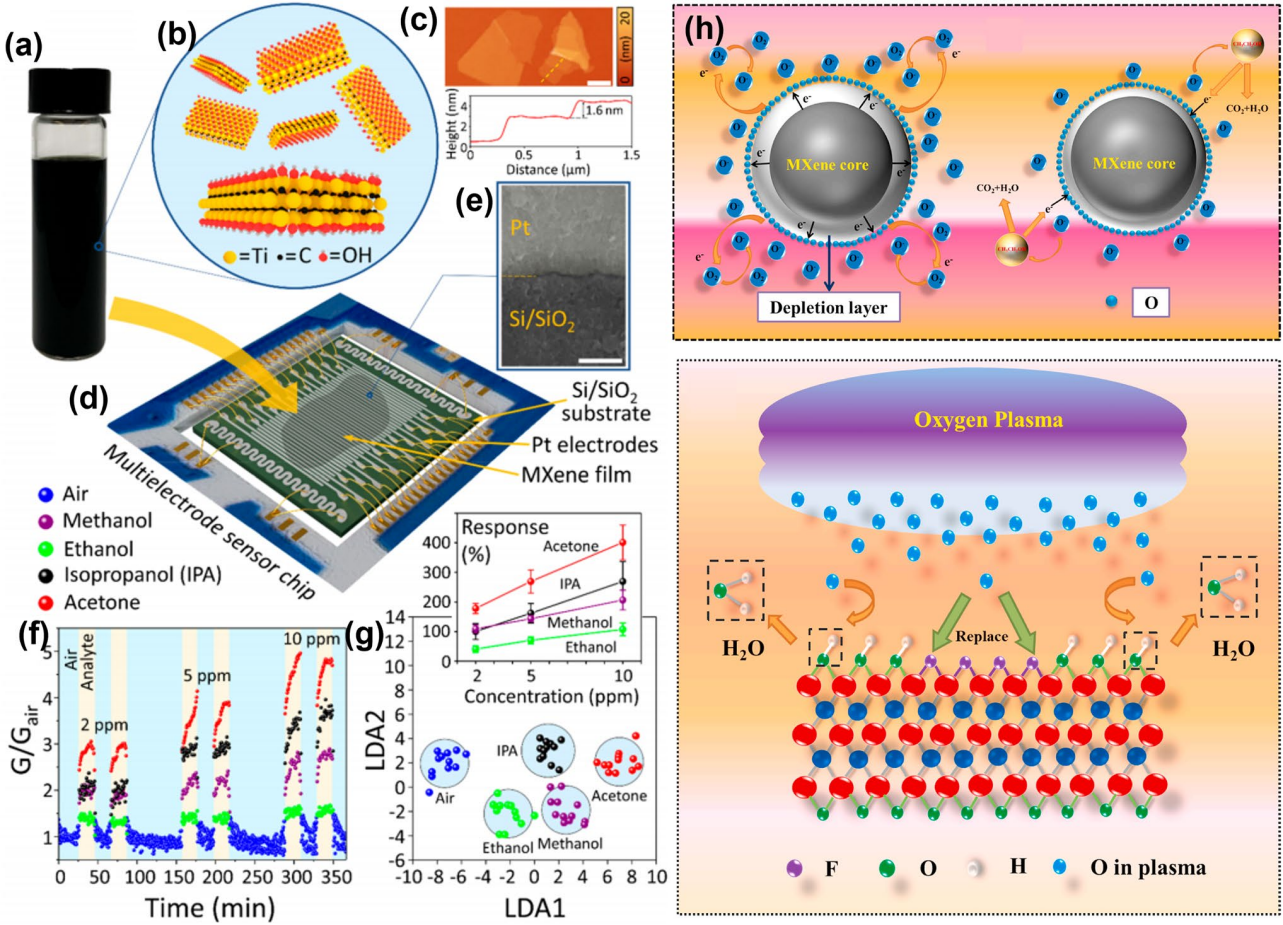
Characterization of gas sensing in partially aluminized Ti_3_C_2_T_x_ MXene films on a multisensor chip. **a** Stabilization of the aqueous solution of layered MXene in photographic form. **b** Diagram of the construction of a single-layer Ti_3_C_2_T_x_ MXene sheet.** c** Photographs of an AFM image of a Ti_3_C_2_T_x_ MXene flake on Si/SiO_2_, the scale bar is 1 μm m. **d** Multi-electrode chip scheme for MXene sheet membrane produced by drop-in casting method.** e** SEM image of the MXene films overlaying the area in contact between the platinum alloy electrode and the Si/SiO_2_ backing. Scale bar is 1 μm. **f** MXene partial conductivity G(t) variation, relative to conductivity in air (in dry air at 350 °C with acetone, isopropyl alcohol (IPA), ethanol and methanol dosed sequentially at 2–10 ppm). **g** Illustration: dependence of chemical reactions, S = ΔG/Gair, average of all MXene sensor elements for organic vapor focus on a chip; error bands indicate the fluctuation of resistance of the entire multisensor array. Reproduced with permission from Ref. [148]. **h** Schematic diagram of the possible gas sensing mechanism of Ti_3_C_2_T_x_ MXene. Reproduced with permission from Ref [149]

**Fig. 9:**
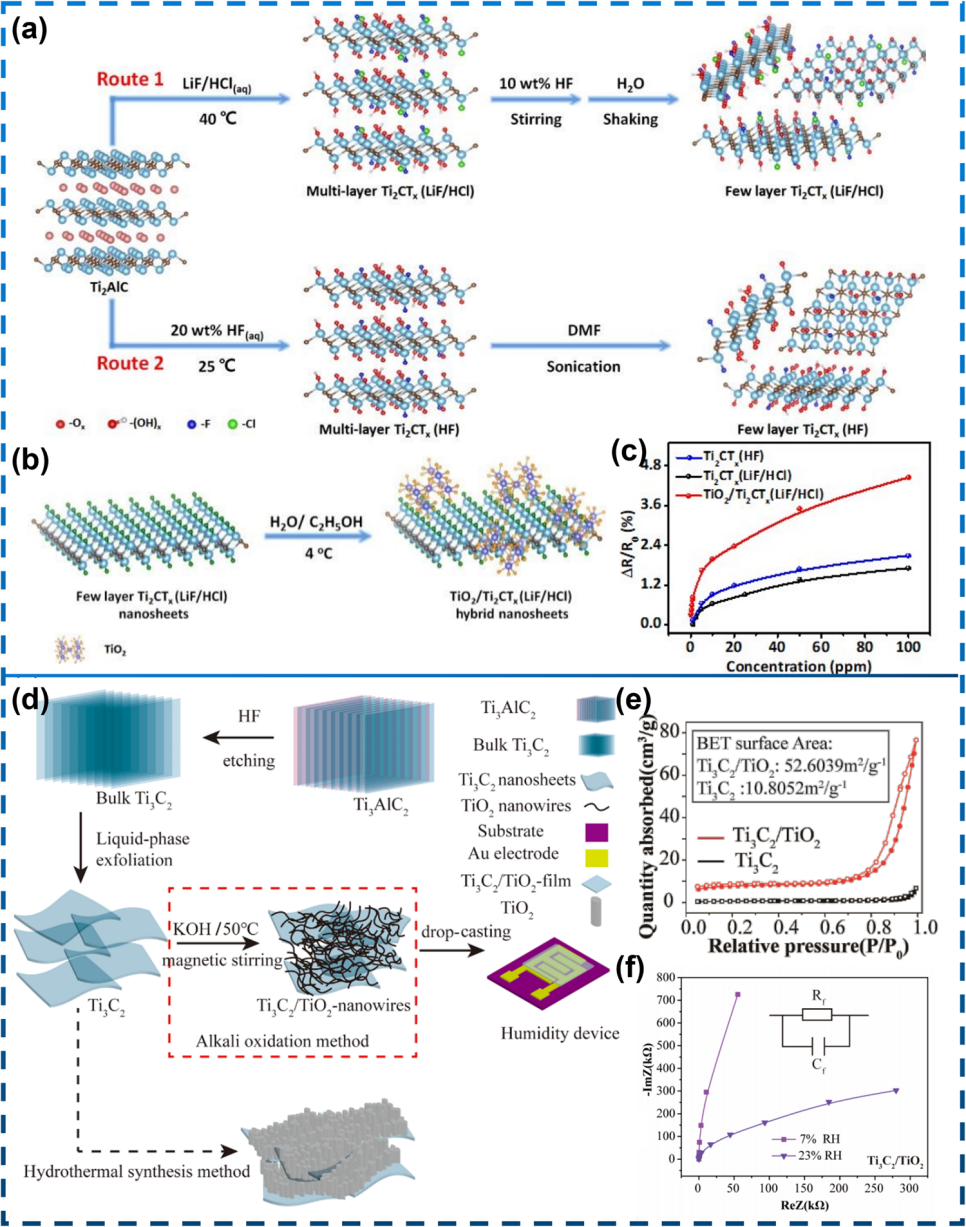
**a** Preparation process of Ti_2_CT_x_ (LiF/HCl) nanosheets (Route 1) and Ti_2_CT_x_ (HF) nanosheets (Route 2) is schematically shown. **b** Diagram of the fabrication of the TiO_2_/Ti_2_CT_x_ (LiF/HCl) blend nanosheets. **c** Regularized resistance changes of each sensor at various NH_3_ levels. Reproduced with permission from Ref. [150]. **d** Ti_3_C_2_/TiO_2_-nanowires material preparation process: HF etching, liquid-phase exfoliation, alkali oxidation, and other methods.** e** BET surface area of Ti_3_C_2_ and Ti_3_C_2_/TiO_2_. **f** Complex impedance plots of Ti_3_C_2_/TiO_2_ composite film at 7%-23% RH. Reproduced with permission from Ref. [159]

In addition to the hydrothermal partial oxidation of Ti_3_C_2_T_x_ mentioned above, researchers have also prepared partially oxidized Ti_3_C_2_T_x_ by heat treatment at 350 °C [148] (Fig. 8a–g) and microwave-activated oxygen plasma [149] treatment (Fig. 8h). Sun et al. [150] investigated the processing-dependent sensing behavior of Ti_2_CT_x_ (LiF/HCl), Ti_2_CT_x_ (HF), and TiO_2_/Ti_2_CT_x_ (LiF/HCl) at room temperature under 365 nm ultraviolet light (Fig. 9a, b). In addition, the results indicate that TiO_2_/Ti_2_CT_x_ (LiF/HCl) exhibits better sensing performance than other samples (Fig. 9c). Since it contains abundant oxygen functional groups (-O_x_, -(OH)_x_ and Ti–O-Ti), providing more NH_3_ molecular interactions. Li et al. [159] developed a humidity sensor by in situ growth of TiO_2_ nanowires on two-dimensional (2D) Ti_3_C_2_ MXene using alkaline oxidation method (Fig. 9d*)*. They found that the sea urchin-like Ti_3_C_2_/TiO_2_ composites have an order of magnitude larger surface area when compared to pure Ti_3_C_2_ or TiO_2_ materials (Fig. 9e) and exhibit documented high sensitivity at environments with low thermal relative humidity (RH) (from 7% RH to 33% RH, approximately 280 pF/% RH) (Fig. 9f).

**Fig. 10:**
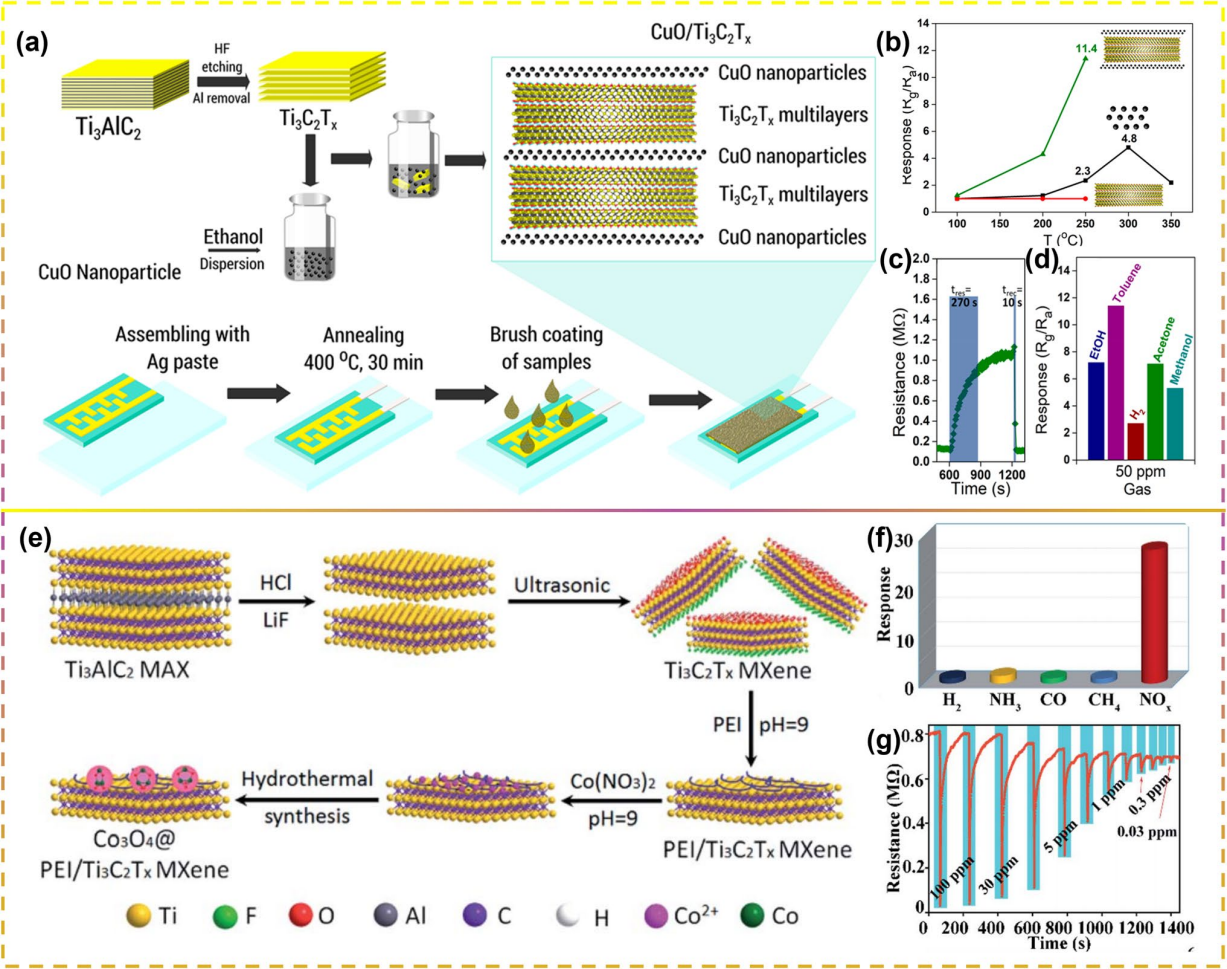
**a** Schematic Representation of a Facile Preparation of CuO nanoparticles/Ti_3_C_2_T_x_ Hybrid Heterostructures and Gas Sensor Device Fabrication.** b** Gas sensing response of CuO, Ti_3_C_2_T_x_ MXene, and CuO/Ti_3_C_2_T_x_ MXene tested at different working temperatures. **c** Response/recovery times. **d** Selectivity of CuO/Ti_3_C_2_T_x_-30 wt% to 50 ppm of tested gas. Reproduced with permission from Ref. [145]. **e** Schematic illustration of the Co_3_O_4_@PEI/Ti_3_C_2_T_x_ MXene composites. **f** CoPM-24 sensor selectivity study under the influence of the presence of various gases at 100 ppm. **g** Momentary feedback of CoPM-24 (Co_3_O_4_@PEI/Ti_3_C_2_T_x_) sensor to 100–0.03 ppm NO_x_. Reproduced with permission from Ref. [146]

CuO exhibits the advantage of wide range response to VOCs, but has the drawbacks of small response values, slow response/recovery speed, and low durability. For this reason, Angga Hermawan et al. [145] reported a simple method to prepare CuO-Ti_3_C_2_T_x_ MXene hybrid by self-assembling electrostatically (Fig. 10a). CuO-Ti_3_C_2_T_x_ MXene showed a better methane gas sensing response (R_g_/R_a_) of 11.4 than pristine CuO nanoparticles at 250°C for 50 ppm toluene gas sensing response nearly five times higher than that of pristine CuO particles for 50 ppm toluene at 250 °C. (Fig. 10b). In addition, due to the high conductivity of the metal phase in Ti_3_C_2_T_x_ MXene, the hybridization of CuO with Ti_3_C_2_T_x_ MXene not only improves the response time, but also improves selectivity, response (270 s), and recovery time (10 s) (Fig. 10c, d).

Sun et al. [146] used simple noncovalent chemical methods and hydrothermal methods to effectively rivet Co_3_O_4_ nanocrystals onto functionalized Ti_3_C_2_T_x_ MXene sheets of branched polyethylene imine (PEI), and prepared Co_3_O_4_@PEI/Ti_3_C_2_T_x_ MXene composite material (Fig. 10e). Sun et al. examined the sensing performance of nitrogen oxides (consisting of NO_2_ and NO) using Co_3_O_4_@PEI/Ti_3_C_2_T_x_ (CoPM) complexes and found that CoPM-24 complexes exhibited 27.9% response when added at 2.4 mg Ti_3_C_2_T_x_ along with high selectivity and very weak detection limits (30 ppb-NO_x_) (Fig. 10f, g). In 2021, Zhang et al. [147] as a high-performance self-powered formaldehyde (HCHO) sensor based on MXene/Co_3_O_4_ composite was prepared. Electricity was supplied through piezoelectric nanogenerators (PENGs) of ZnO/MXene nanowire arrays. p-type metal oxide Co_3_O_4_ provided more active sites for formaldehyde interactions, thus the MXene/Co_3_O_4_ composite exhibited good response at room temperature with 9.2% response at 10 ppm HCOH and low detection limit—0.01 ppm.

**Fig. 11:**
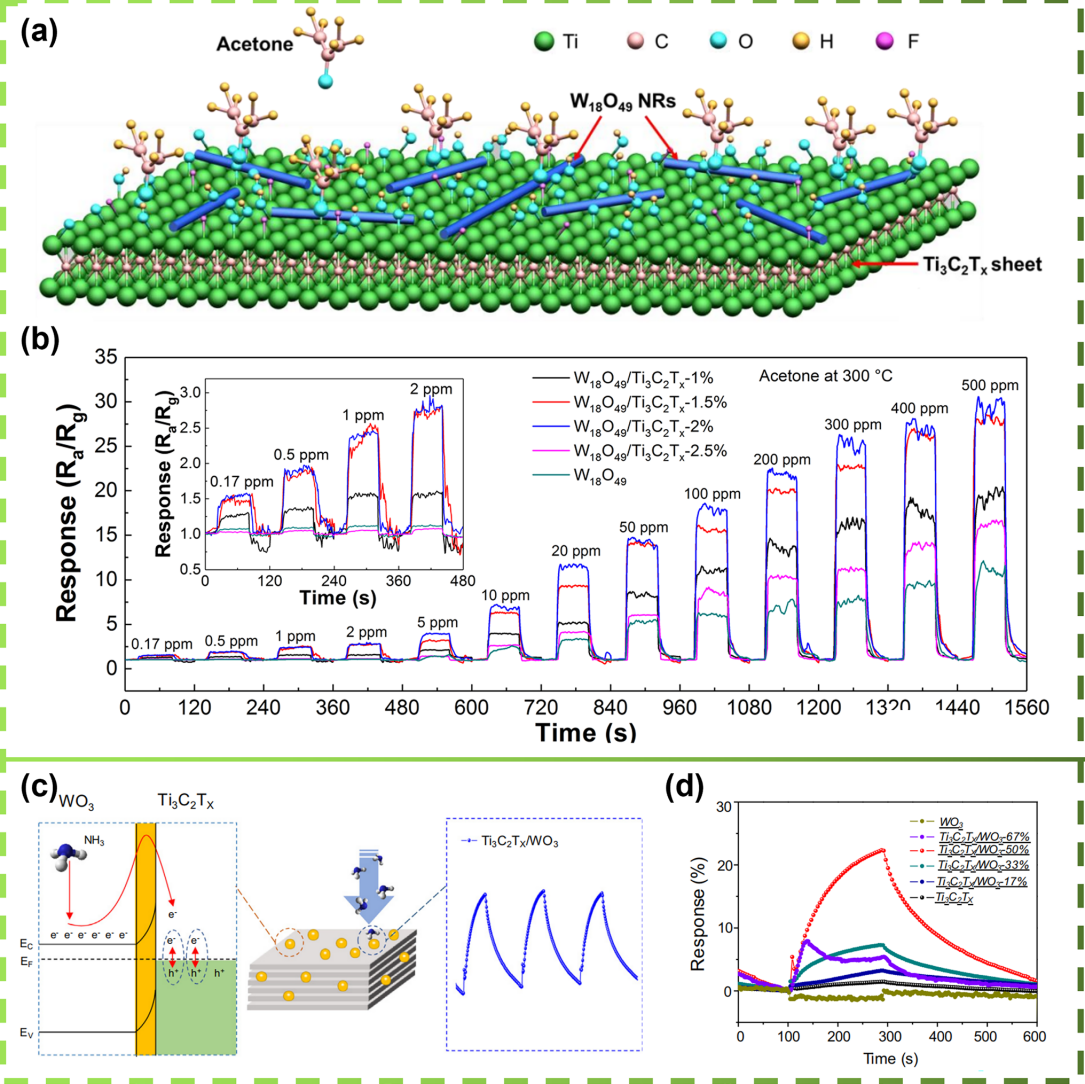
**a** Image demonstrating the mechanism of acetone sensing by W_18_O_49_/Ti_3_C_2_T_x_ nanocomposites. **b** Image of instantaneous response curves of W_18_O_49_/Ti_3_C_2_T_x_-based sensors in the range of acetone concentrations from 0.17 to 500 ppm. Reproduced with permission from Ref. [187]. **c** Mechanisms of sensing of NH_3_ by Ti_3_C_2_T_x_/WO_3_-50% of composites. **d** Different amounts of WO_3_ of the composite sensor in response behavior to 1 ppm NH_3_ at room temperature. Reproduced with permission from Ref. [151]

In 2019, Sun et al. [187] used a simple solvothermal method to grow one-dimensional W_18_O_49_ nanorods in situ on the Ti_3_C_2_T_x_ surface. The W_18_O_49_/Ti_3_C_2_T_x_ composites exhibited a high response to low concentrations of acetone (11.6–20 ppm) (Fig. 11a), as well as high selectivity, long-term stability, and also a fast response and recovery response to very low of acetone (170 ppb) can also be detected (Fig. 11b). Physical properties of NH_3_ sensing were improved by forming heterojunctions and enhancing the number of active sites, relative surface area, and pore size of pristine Ti_3_C_2_T_x_ by functionalizing Ti_3_C_2_T_x_ with WO_3_ nanoparticles by a simple ultrasonic method, as shown in Fig. 11c. The resulting Ti_3_C_2_T_x_/WO_3_-50% (weight percent of WO_3_) sensor exhibited excellent response to NH_3_ (22.3% at 1 ppm), which was 15.4 times that of the pristine Ti_3_C_2_T_x_ sensor (1.54% at 1 ppm) with no electrical resistance drift (Fig. 11d) [151].

**Fig. 12:**
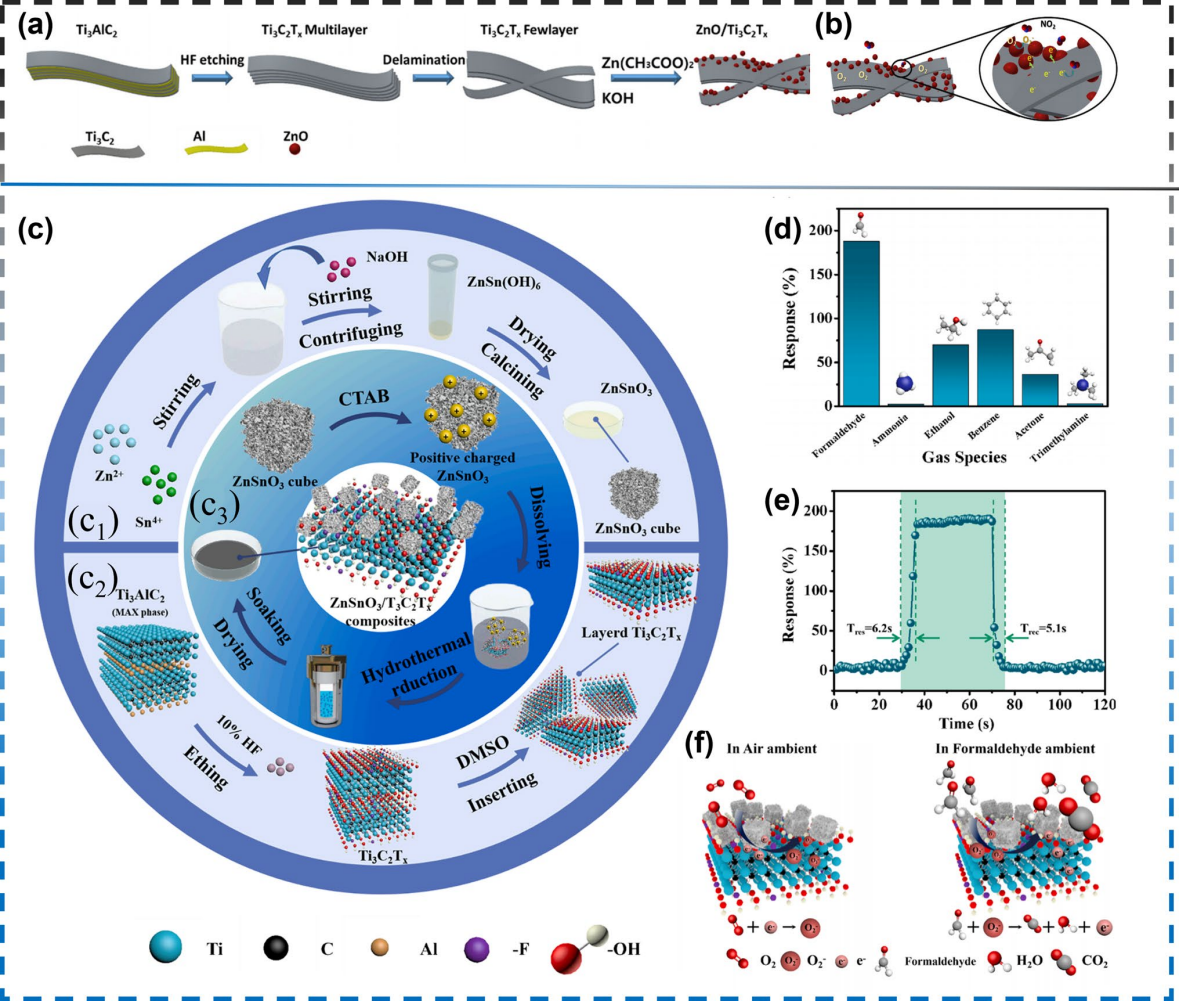
**a** Schematic synthesis procedure of ZnO/Ti_3_C_2_T_x_ heterostructure.** b** Schematic NO_2_-sensing reaction mechanism of ZnO/Ti_3_C_2_T_x_ nanocomposite. Reproduced with permission from Ref. [157]. **c** Schematic of fabrication process of (c_1_) ZnSnO_3_ nanocube, (c_2_) layered Ti_3_C_2_T_x_ MXene and (c_3_) ZnSnO_3_/Ti_3_C_2_T_x_ MXene composites. **d** Selective curve of ZnSnO_3_/Ti_3_C_2_T_x_ MXene composites to 100 ppm different gas at room temperature.** e** Response performance of ZnSnO_3_/Ti_3_C_2_T_x_ MXene composites to 100 ppm form aldehyde at room temperature. **f** Schematic of gas sensing mechanism of ZnSnO_3_/Ti_3_C_2_T_x_ MXene composites. Reproduced with permission from Ref. [158]

In addition to this, He et al. [152] successfully synthesized two-dimensional (2D) MXene modified by tin dioxide nanoparticles for gas sensing detection as well by hydrothermal method. Wang et al. [153] successfully synthesized SnO-SnO_2_ (p–n junction) and Ti_3_C_2_T_x_ MXene nanocomposites for gas sensing by a one-step hydrothermal method. Zinc oxide (ZnO) has long been used as a gas detector. Although it has good response to various gases, high operating temperature limits the widespread application as a gas sensing material. Qui et al. [157] ZnO/Ti_3_C_2_T_x_ MXene nanocomposite composed of 2D multilayer MXene and 1D ZnO nanoparticles prepared a room temperature toxic gas sensor (Fig. 12a). The nanocomposite material exhibits enhanced response and recovery behavior to toxic gases, superior to pure Ti_3_C_2_T_x_ MXene and pure ZnO. Its gas sensing principle is shown in Fig. 12b. Under the irradiation of the sun, ZnSO_3_ nanocube and layered Ti_3_C_2_T_x_ MXene were synthesized by simple static self-assembly to synthesize ZnSO_3_/Ti_3_C_2_T_x_ MXene nanocomposites (Fig. 12c). Sima et al. [158] found that the ZnSO_3_/Ti_3_C_2_T_x_ MXene nanocomposite-based sensor displayed significant selectivity for formaldehyde, with high response (194.7% to 100 ppm and 62.4% to 5 ppm) (Fig. 12d), and rapid response/recovery times (6.2/5.1 s at 100 ppm formaldehyde) (Fig. 12e), and these tests were conducted at RT. Figure 12f shows the gas sensing scheme of the ZnSO_3_/Ti_3_C_2_T_x_ MXene laminate.

**Fig. 13:**
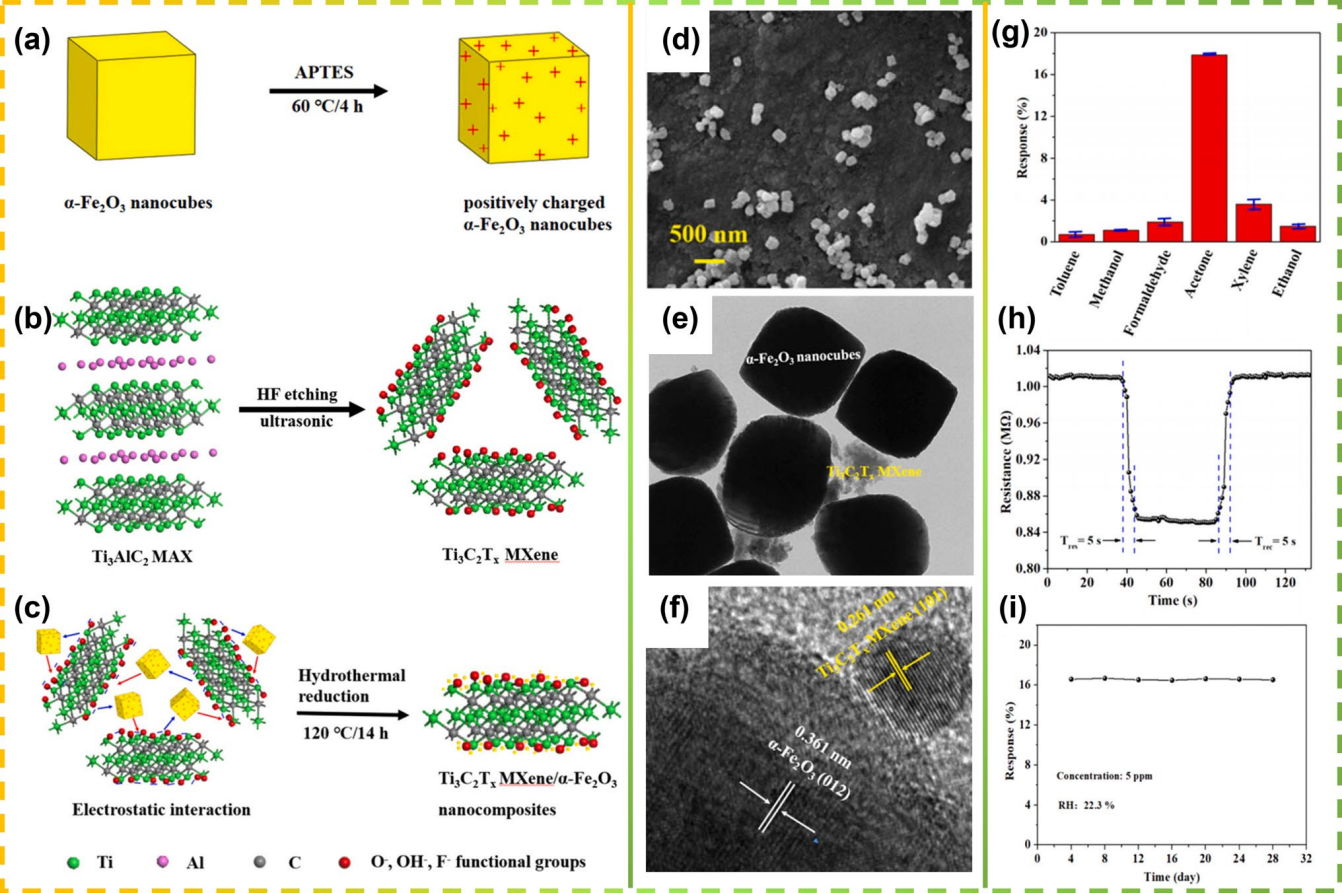
Illustration of the preparation process of **a** positively charged α-Fe_2_O_3_ nanocubes, **b** Sheet-like Ti_3_C_2_T_x_ MXene and **c** α-Fe_2_O_3_/Ti_3_C_2_T_x_ MXene composites. **d** SEM images of α-Fe_2_O_3_/Ti_3_C_2_T_x_ MXene composites. **e** TEM image. **f** HRTEM image of the α-Fe_2_O_3_/Ti_3_C_2_T_x_ MXene composites. Reproduced with permission from Ref. [155]. **g** Selective property of the sensor based on α-Fe_2_O_3_/Ti_3_C_2_T_x_ MXene composites to 5 ppm of various target gases at room temperature. **h** The real-time resistance measurement of α-Fe_2_O_3_/Ti_3_C_2_T_x_ MXene composite sensor toward acetone vapor at RT. **i** Long-term stability of the α-Fe_2_O_3_/Ti_3_C_2_T_x_ MXene-based sensor for 5 ppm acetone. Reproduced with permission from Ref. [160]

Different research groups have conducted extensive research on gas sensing performance using pure Ti_3_C_2_T_x_ and its complexes with different metal oxides, such as V_2_O_5_ [45], $\alpha$-Fe_2_O_3_ [155], In_2_O_3_ [156], and K_2_Ti_4_O_9_ [160]. Liu [155] successfully prepared heterogeneous composite materials of $\alpha$-Fe_2_O_3_ and Ti_3_C_2_Tx MXene using a simple hydrothermal method (Fig, 13a-c), and characterized their morphology and microstructure through various characterization methods (Fig. 13b–f). The results indicate that a size of approximately 250 nm wide was prepared $\alpha$-Fe_2_O_3_ nanocube and uniformly distributed on the surface of Ti_3_C_2_T_x_ MXene nanosheets. The results indicate that a size of approximately 250 nm wide was prepared $\alpha$-Fe_2_O_3_ nanocube and uniformly distributed on the surface of Ti_3_C_2_T_x_ MXene nanosheets. The gas sensitivity test results show that compared with other typical gases, the sensor based on $\alpha$-Fe_2_O_3_/Ti_3_C_2_T_x_ MXene composite material exhibits excellent selectivity toward acetone, and very favorable response to 5 ppm acetone: 16.6% (Fig. 13g), high rate of response and recovery: 5/5 s (Fig. 13h), excellent linearity, and significant repeatability at room temperature (RT) (Fig. 13i) [160].

Taken together, as shown in Table 3, TiO_2_ is the most commonly used metal oxide composition to assist MXenes in detecting reducing gases at room temperature. For the response of NH_3_ gas, MXenes, and tungsten tin oxides showed the best response values, but the lower response limit did not change significantly, and the response recovery time needs further investigation [164, 192]. On the other hand, we found that Co_3_O_4_ and ZnO are suitable support materials for the detection of oxidizing gases using MXenes. In the presence of In_2_O_3_ and $\alpha$-Fe_2_O_3_, other reducing volatile organic compounds, such as methanol and acetone, were better perceived, respectively.

### MXene/TMDs

**Fig. 14:**
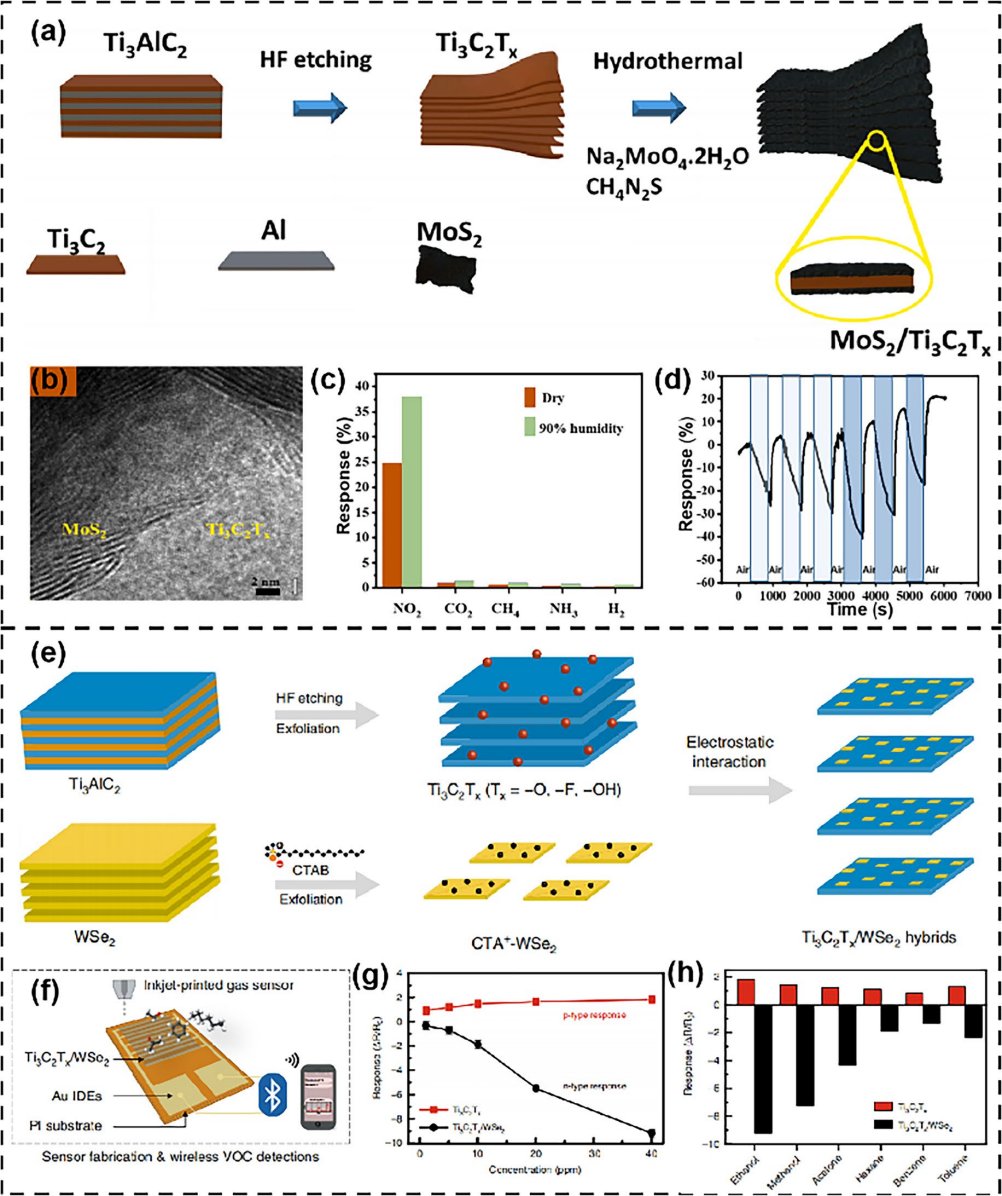
**a** Schematic illustrating the synthesis process of the MoS_2_/Ti_3_C_2_T_x_ heterostructure from the Ti_3_AlC_2_ MAX phase. **b** HRTEM images of the MoS_2_/Ti_3_C_2_T_x_ heterostructure. **c** Comparison of responses of MT2 sample to various gases at 10 ppm concentration. **d** Cyclic responses of MT2 to 10 and 20 ppm NO_2_ gas. Reproduced with permission from Ref. [161]. **e** Schematic illustration of preparation processes for Ti_3_C_2_T_x_/WSe_2_ nanohybrids. **f** Schematic illustration of inkjet-printed gas sensors in detection of volatile organic compounds with a wireless monitoring system. **g** Comparison of gas response as a function of ethanol gas concentrations for Ti_3_C_2_T_x_ and Ti_3_C_2_T_x_/WSe_2_ sensors. **h** Selectivity test of the Ti_3_C_2_T_x_ and Ti_3_C_2_T_x_/WSe_2_ sensors upon exposure to various VOCs at 40 ppm. Reproduced with permission from Ref. [162]

Two-dimensional chalcogenides are two-dimensional materials with unique structures, excellent mechanical, electrical, optical properties, and low energy consumption. It is a well-explored sensing application material. However, for gas/VOC sensing, the research on the composite materials of 2D chalcogenides and MXene is still a rarely explored field. So far, there are only two reports on the combination of MXene and sulfides for gas sensing. Firstly, Qui et al. [161] prepared MoS_2_/Ti_3_C_2_T_x_ heterostructures with interconnected network nanostructures through a simple hydrothermal method (Fig. 14a). The synthesized MoS_2_/Ti_3_C_2_T_x_ heterostructure exhibits significant lattice matching (Fig. 14b), where vertically arranged MoS_2_ nanosheets grow on Ti_3_C_2_T_x_ MXene and have a large specific surface area. The obtained gas sensor exhibits very high sensitivity and selectivity to NO_2_ gas exposure, reaching up to 25% at 10 ppm, as well as rapid recovery and long-term stability (Fig. 14c, d). Due to the large number of Mo active sites and the conductivity of Ti_3_C_2_T_x_ MXene, which can accelerate electron movement and excellent heterojunction interface contact, the presented structure exhibits enhanced NO_2_ sensing activity. Secondly, Chen et al. [162] reported on the Ti_3_C_2_T_X_/WSe_2_ nanohybrid material, which was prepared through simple surface treatment and peel-based process (Fig. 14e), and combined as a sensing material into inkjet printing and wireless operation sensors (Fig. 14f). The sensing measurement has excellent repeatability and reproducibility. The energy band diagram of the Ti_3_C_2_T_x_/WSe_2_ sensor in the presence of ethanol shows n-type sensing behavior and Schottky barrier modulation (Fig. 14g). Compared with sensors made from raw Ti_3_C_2_T_x_ and raw WSe_2_, the Ti_3_C_2_T_x_/WSe_2_ hybrid sensor exhibits a 12-fold improvement in ethanol sensitivity, low electrical noise, sound selectivity, and ultra-fast response/recovery characteristics (Fig. 14h). Table 3 summarizes a detailed overview of sensors for MXene and TMDs composite materials.

### MXene/MOF

**Fig. 15:**
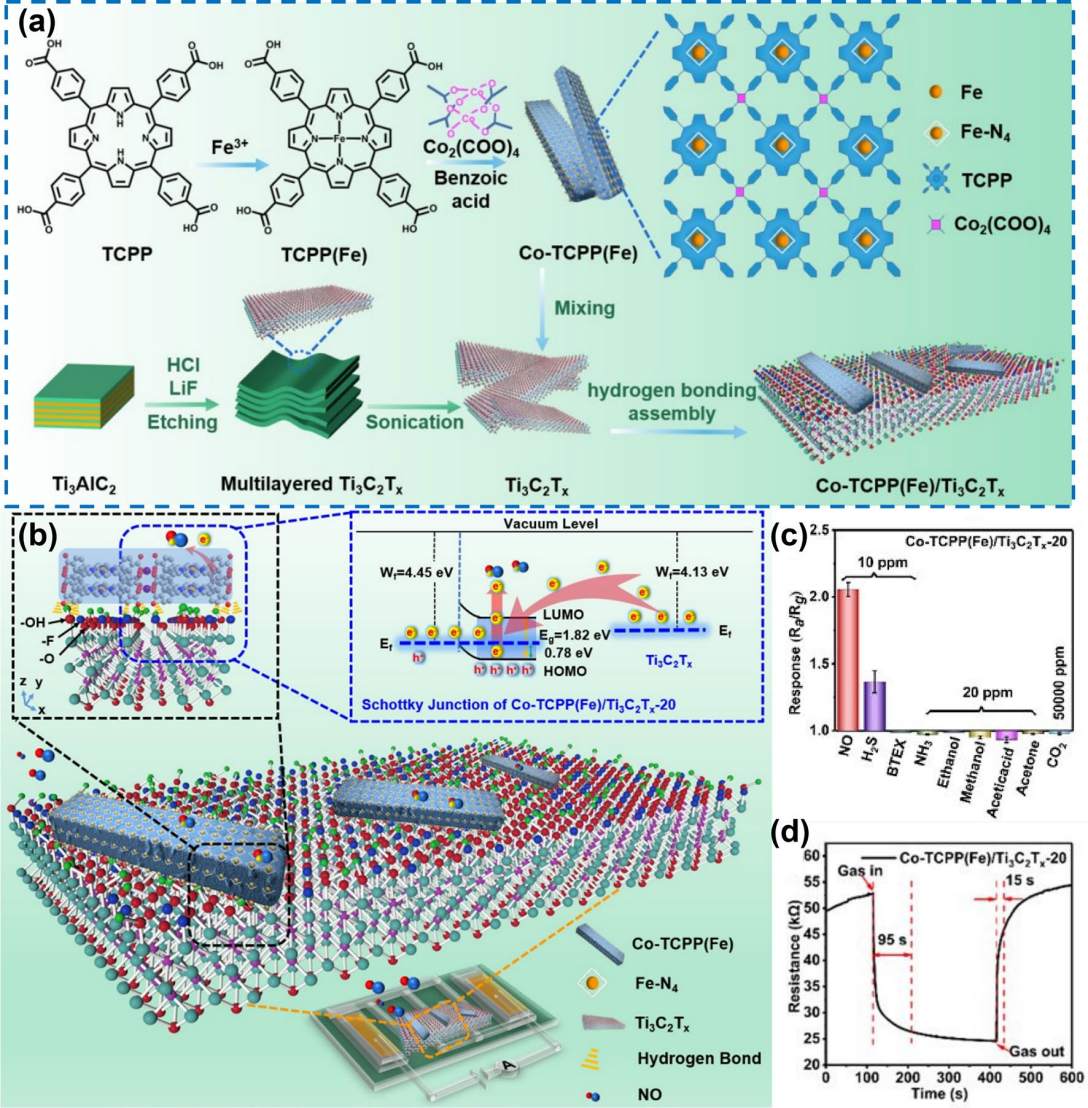
**a** Synthesis process of Co-TCPP(Fe), Ti_3_C_2_T_x_, and Co-TCPP(Fe)/Ti_3_C_2_T_x_. **b** Schematic diagram of the sensing mechanism of the Co-TCPP(Fe)/Ti_3_C_2_T_x_-20 toward NO. **c** Selectivity of the sensor to various gases at concentrations of 10 and 20 ppm. **d** Real-time response–recovery curve of the Co-TCPP(Fe)/Ti_3_C_2_T_x_-20 based sensor toward 10 ppm NO at room temperature. Reproduced with permission from Ref. [164]

In recent decades, metal organic frameworks (MOFs) have developed rapidly and their popularity has not decreased, making them a hot topic in the field of materials. However, the conductive MOF obtained by combining MOF and MXene breaks the shackles of MOF materials that are almost non-conductive, perfectly combines the controllable structure of organic materials and the long-term order of inorganic materials, plus the unique high electron mobility, conductive MOF can be described as a favorite, and is also one of the most potential materials in gas sensing applications [163], such as Chang et al. [164] designing and preparing a rod-shaped porphyrin based metal oxide (Co TCP (Fe)) and MXene (Ti_3_C_2_T_x_) through hydrogen bonding to form a chemically resistant NO sensing hybrid (Co-TCPP (Fe)/Ti_3_C_2_T_x_) (Fig. 15a). The sensor based on Co TCP (Fe)/Ti_3_C_2_T_x_ shows excellent NO sensing performance at room temperature (Fig. 15b), including high response (= 2.0, 10 ppm) (Fig. 15c), reliable repeatability, high selectivity, low actual detection limit (pLOD, 200 ppb), and rapid room temperature NO sensing response/recovery speed (95/15 s, 10 ppm) (Fig. 15d).

### MXene/Polymer

**Fig. 16:**
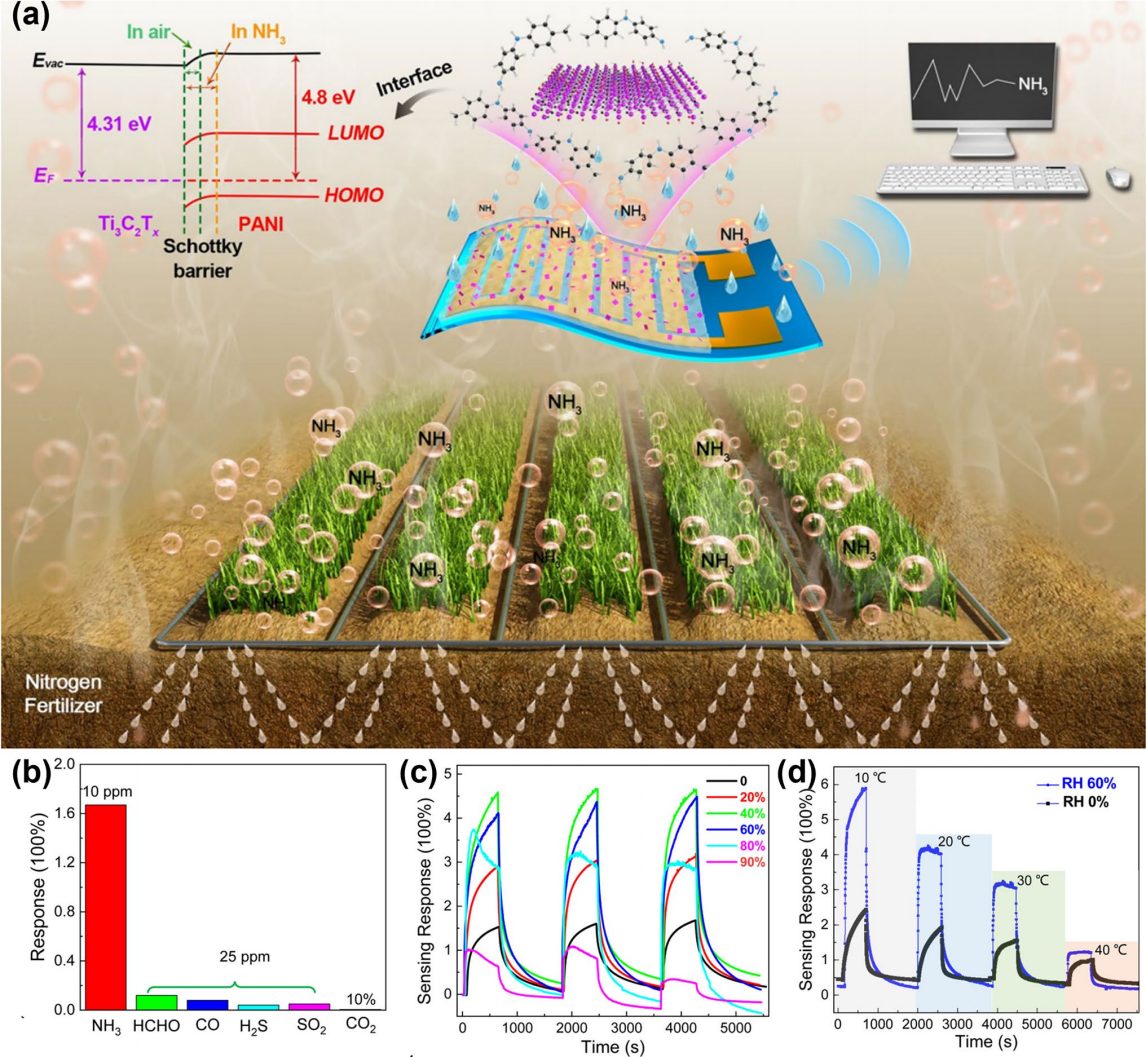
**a** Application scenarios of PANI/Ti_3_C_2_T_x_ hybrid sensitive film-based flexible NH_3_ sensor for ammonia volatilization monitoring in agriculture. **b** Selectivity of the hybrid sensor to NH_3_ and other interference gases in agricultural fields at room temperature. **c** Moisture dynamic response of the NH_3_ sensing performance of PANI/Ti_3_C_2_T_x_ hybrid sensitive films. **d** Dynamic sensing response of the hybrid sensor toward 10 ppm NH_3_ in the range of 10–40 °C at dry air and 60% RH. Reproduced with permission from Ref. [165]

**Fig. 17:**
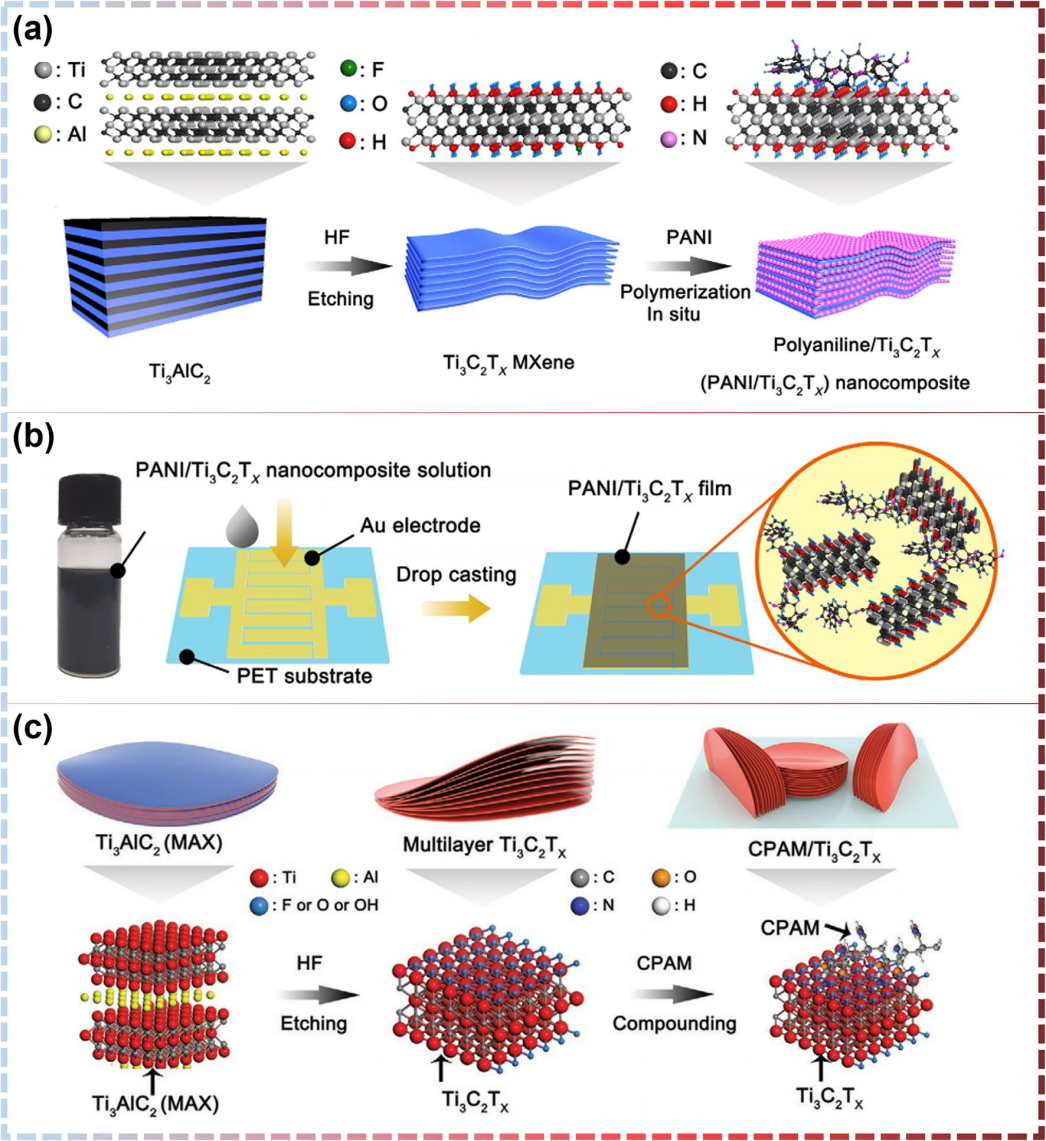
**a** A diagram of the composite synthesis of PANI/Ti_3_C_2_T_x_ nanocomposite, which includes the peeling process of Ti_3_AlC_2_ and the consolidation process of ANI. **b** Sketch of the Inter-digital polarization of the electrodes shown before and after plating PANI/Ti_3_C_2_T_x_ nanocomposites. Reproduced with permission from Ref. [105]. **c** Synthesis scheme of CPAM/Ti_3_C_2_T_x_ nanocomposites, including the etching process for Ti_3_AlC_2_ and composite process of CPAM and Ti_3_C_2_T_x_. Reproduced with permission from Ref. [166]

Polymers have excellent flexibility, favorable sensitivity, appropriate electrical conductivity, low cost, a large number of organic groups to interact with the gas on the surface, light weight, and low reaction temperature, making them suitable for gaseous/VOC sensing applications when mixed with MXenes. MXene/polymer composite sensors are used to identify ammonia [101, 103, 165, 166], ethanol [32, 105], methanol [33, 104], acetone [31, 102], and humidity [99, 100, 112, 113] for wear and tear [189–194]. With respect to ammonia identification, the original MXene-based sensor shows excellent NH_3_ sensing characteristics, but ammonia has very high adsorption energy and NH_3_ is difficult to partition from the MXene screen during recovery, and demonstrates extended recognition time as well as wandering of the baseline resistance. To surmount these limitations, Li et al. [165] developed in situ a flexible chemorepulsive gas sensor based on a hybrid polyaniline (PANI)/Ti_3_C_2_T_x_ sensitive layer for tracking ammonia volatilization out of agriculture using self-assembled method in situ (Fig. 16a). The sensor exhibits excellent NH_3_ sensing performance over a temperature range of 10–40°C at 20%–80% relative humidity (RH) (sensing response to 10 ppm ammonia peaks at 4.7 at 40% RH, which is almost three times higher than in dry air (~ 1.6)) (Fig. 16b–d). Zhao et al. [105] also used over-PANI, via a low-temperature in situ polymerization method to rationally modified PANI particles coated with Ti_3_C_2_T_x_ nanosheets (Fig. 17a, b). This evoked remarkable detection sensitivity, a rapid response/recovery rate and mechanistic stability as well at room temperature. A year later, Zhao et al. [166] also developed room temperature nanocomposites based on 2D MXenes materials and cationic polyacrylamide (CPAM) (Fig. 17c) with high gas responsiveness and flexibility aimed at building high-performance ammonia sensors.

**Fig. 18:**
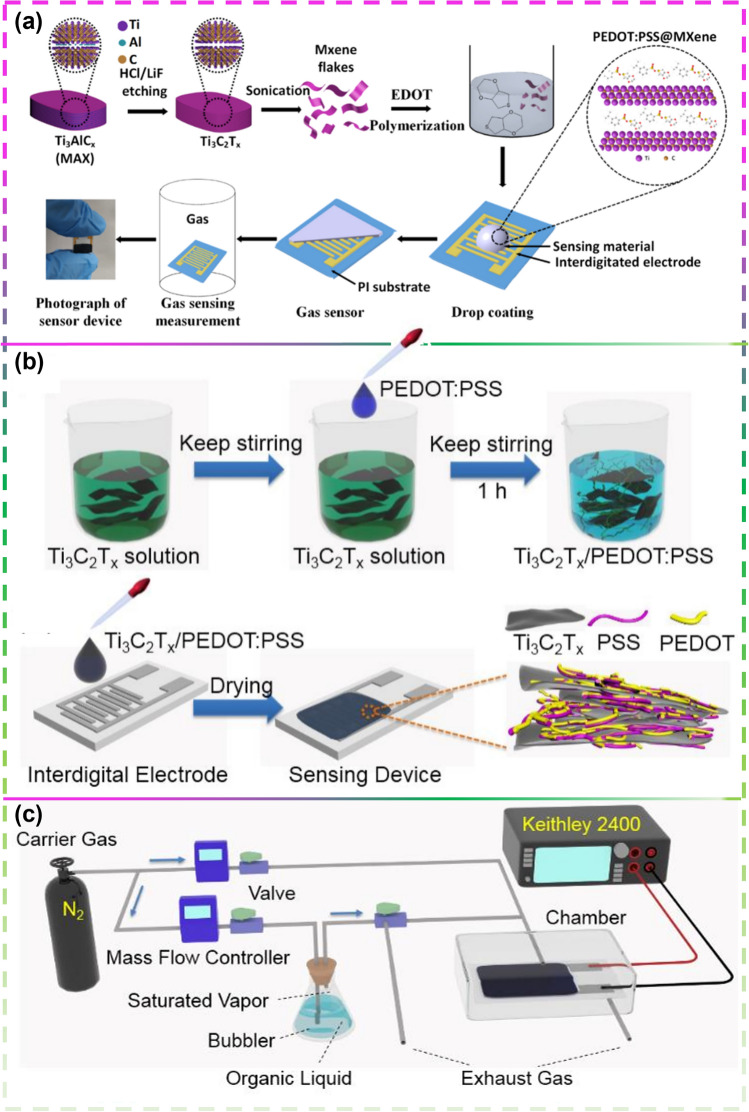
**a** Schematic Illustration for the Synthesis of PEDOT:PSS/MXene Composites and the Fabrication Process of the Composite-Based Gas Sensor. Reproduced with permission from Ref. [103]. **b** Ti_3_C_2_T_x_/PEDOT:PSS profile of material and gas sensor manufacturing. **c** The diagram of the experimental setup. Reproduced with permission from Ref. [104]

Conductive polymers-3,4-ethylenedioxythiophene (EDOT) and poly(4-styrenesulfonate) (PSS) are also commonly used to composite with MXene. Jin et al. [103] used a dip coating technique to make a gas sensor from the resulting PEDOT:PSS/MXene composite (Fig. 18a). NH_3_ at room temperature demonstrated a strong gas response of 36.6% to 100 ppm NH_3_ with recovery and response times of 116 and 40 s. Furthermore, the hybrid sensor presented stronger sensitivity performance compared to pure PEDOT:PSS and Ti_3_C_2_T_x_ MXene-based sensors, evidencing that the PEDOT:PSS copolymer and Ti_3_C_2_T_x_ MXene two-dimensional ingredients have a synergistic effect on each other. In addition to showing a high response to ammonia gas, it also responded well to other gases, e.g., Wang et al. [104] used a 4:1 mixture of PEDOT:PSS and Ti_3_C_2_T_x_ to prepare a methanol gas sensor (Fig. 18b, c), where the reaction rate of 5.54 was high for the largest reaction and the second largest reaction tested at room temperature when compared to pure PEDOT:PSS and pure Ti_3_C_2_T_x_.

**Fig. 19:**
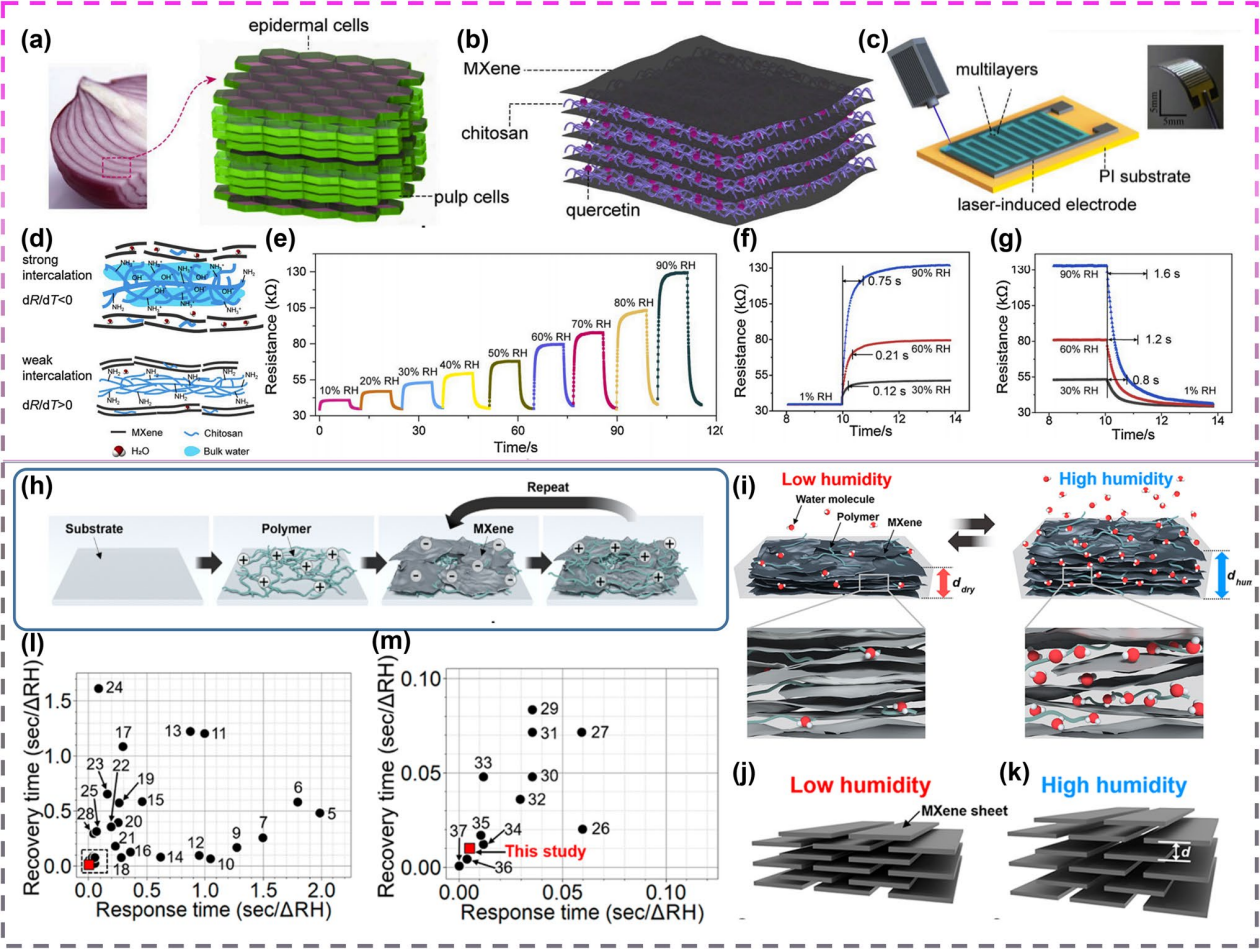
**a** Photograph of purple onion scale leaves and schematic diagram of the scale leaves. **b** Schematic diagram of the MCQMs composed of MXene flakes and chitosan–quercetin membranes. **c** The humidity sensor based on laser-induced interdigitated electrode upon PI substrate. Inset shows the photograph of the flexible humidity sensor. **d** Chitosan and H_2_O intercalation induced by MCQMs. **e** 4-Layer induction response to MCQMs. **f and g** A study of the reaction/recovery time of four layers to MCQM under diverse humidity conditions. Reproduced with permission from Ref. [112]. **h** Schematic of the PDAC/MXene assembly. **i** Schematic illustrations showing the proposed humidity response mechanism of the MXene/polyelectrolyte multilayers. Schematic diagrams of MXene/polyelectrolyte multilayers and the corresponding electrical circuit models for **j** low and **k** high humidity. **l** and** m** Comparison of recovery and response times between the MXene/polyelectrolyte multilayers from this study and other humidity sensors reported in the literature. Reproduced with permission from Ref. [110]

For humidity sensing, composites of polymers with MXene are excellent materials. The synergistic effect of chitosan-modified Ti_3_C_2_T_x_ exhibited remarkable performance, enhancing the electrical response to H_2_O molecules. Inspired by the structure of onions (Fig. 19a), Li and colleagues [112] synthesized ion-excited MXene/chitosan–quercetin multilayer membranes (MCQMs) using a layering-by-layer assembly approach (Fig, 19b, c) for which strong interactions to the molecules were found (Fig. 19d). The monolayer pair exhibited the highest resistance in MCQMs, with improved conductivity and reproducibility as the number of layers increased, and the sensor exhibited an ultra-high responsiveness (317% at 90% RH), a wide field of detection, and praiseworthy response and recovery speeds (0.75 and 1.6 s at 90% RH) (Fig. 19e–g). For true breathing studies, An et al. [110] described the mechanism of aqueous adsorption of a multilayer component made of MXene microsheets with polyelectrolytes (Fig. 19h) intended for super-fast humidity sensing (Fig. 19i–k), and they showed that MXene/polyelectrolyte multilayers prepared using layer-by-layer (LbL) components exhibited response and recovery times exceeding those of most humidity sensors (Fig. 19l, m).

In addition, comparing all MXene/polymer gas sensing materials in Table 3, it was found that among all reported polymers, PEDOT: PSS and polyaniline were the most suitable for improving NH_3_ sensing at room temperature together with MXenes. The biopolymer cellulose composite with MXenes (Ti_3_C_2_T_x_/PANI/bacterial cellulose) was the most suitable for humidity sensing [195–208]. The main advantages of polymer doping with MXene are the improved selectivity and sensitivity of MXene, the disadvantages of which are poor stability and more stringent environmental requirements during measurements [148, 209–215].

### Other Materials

**Fig. 20:**
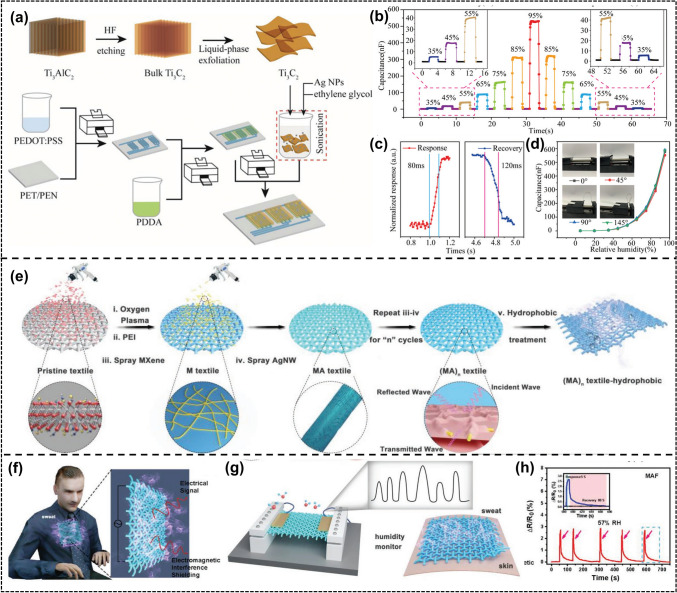
**a** Flowsheet for the fabrication of Ti_3_C_2_/Ag-based moisture sensor by inkjet publishing method. **b** Characteristics of the TA2 response and recovery of the sensor exposed to varying relative humidity (RH) conditions. **c** Duration of response and recovery of sensor TA2. **d** Application of sensor TA2 to various curvature measurement performance. (TA2: Ti_3_C_2_/Ag = 2wt%). Reproduced with permission from Ref. [111]. **e** Schematic illustrating the fabrication of hydrophobic, permeable, and conductive silk textile with a vacuum-assisted layer-by-layer assembly approach.** f** Schematic of the MAF silk detecting sweating humidity. **g** Humidity response of (MA)_n_F silk for monitoring human sweating. **h** Sensitivities of electrical resistance change at 57% RH for MAF silk. (MAF: MXene/Ag NWs/POTs). Reproduced with permission from Ref. [108]

**Fig. 21:**
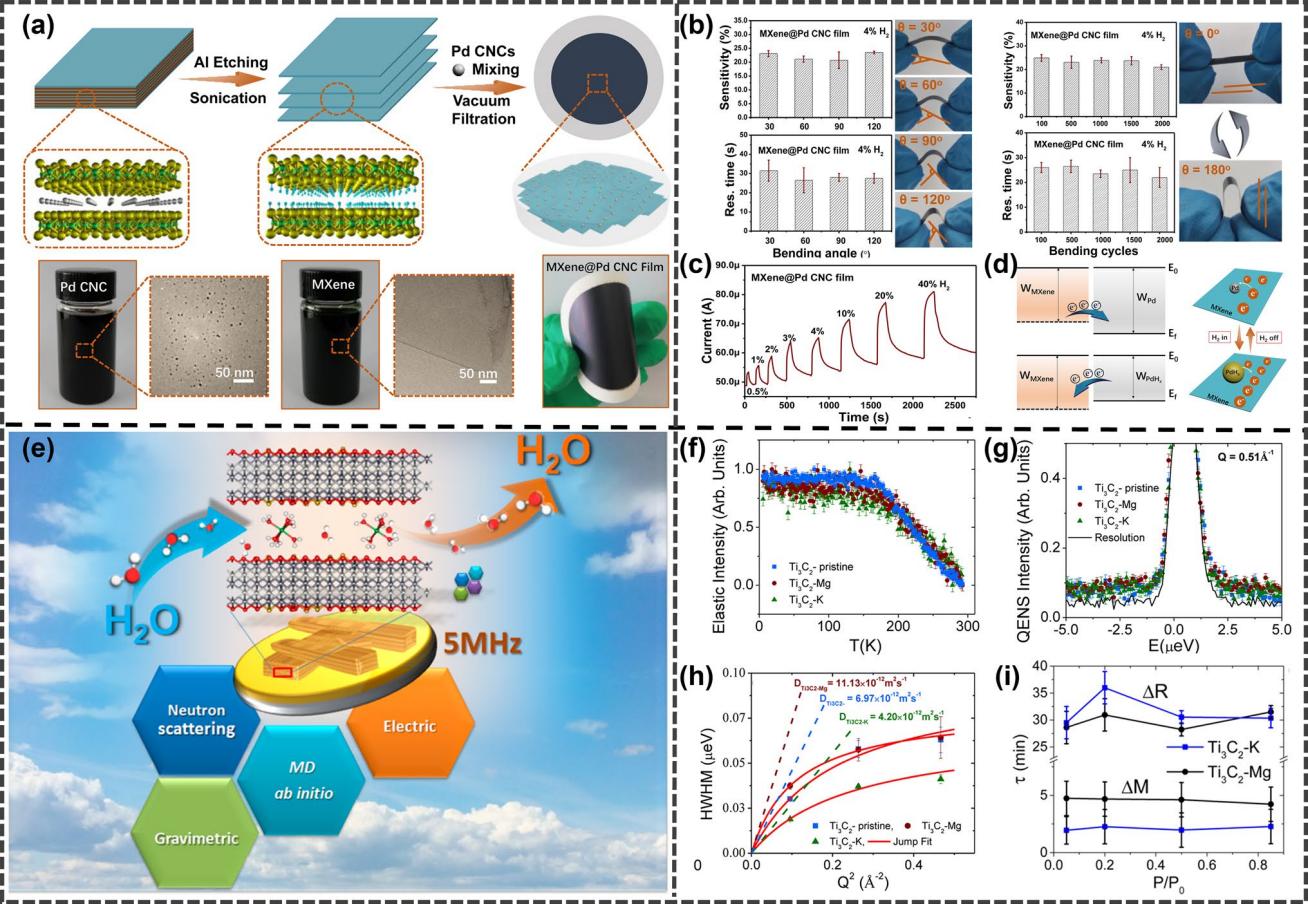
**a** Diagrams of the manufacturing of MXene and MXene@Pd CNC films and photographs of the completed Pd CNC and MXene suspending solutions. **b** Sensitivities and response times of MXene@Pd CNC film sensor to 4% H_2_ (left) and the corresponding flexibility show (right) under different bending angles. Sensitivities and response times of MXene@Pd CNC film sensor to 4% H_2_ after n-time bending cycles (left) and one bending cycle show from $\theta$=0° to 180° and back to 0°. **c** MXene@Pd CNC film real-time response/recovery profiles for a wide range of high H_2_ compositions (0.5 ~ 40 v/v%). **d** Band diagrams of Pd and MXene before and after being exposed to H_2_, and electronic transfer between the surface H_2_ sorbed and Pd CNC and MXene. Reproduced with permission from Ref. [97]. **e** Design the structure of MXenes interaction between water vapor and ion insertion. **f** The normalized elastic strength of mature MXene samples measured at 2 K increments over a temperature interval of 20 to 300 K. **g** A representative normalized QENS spectrum was measured at 300 K from the same sample with a representative Q = 0.51 Å^−1^. **h** Dependence of half-width at half-maximum extracted from the model fit on Q^2^ Solids lines are jump diffusion model fits. The extracted water diffusion coefficient values are shown. **i** Elastic time constants for the reactions of $\Delta$ R and $\Delta$ M during H_2_O desorption ($\tau$). Reproduced with permission from Ref. [110]

Li et al. [111] fabricated a transparent mobile hygrometer using an inkjet printing technique, using a Ti_3_C_2_/Ag blend as a humidity-sensitive membrane and polydiallyldimethylammonium chloride-based (PDDA) as an adhesive barrier (Fig. 20a). The sensor has ultra-high sensitivity (106 ± 800%) (Fig. 20d), fast responsiveness (80 ms), and excellent resistance to bending (Fig. 20c, d). Liu et al. [105] reported a vacuum-assisted layer-by-layer assembly technique (Fig. 20e) for conformal deposition of conductive materials on textiles (Fig. 20f, g), resulting in a leaf like nanostructure composed of silver nanowires (AgNWs) as high conductivity skeletons (veins) and transition metal carbide/carbon nitride (MXene) nanosheets as thin layers. Having a highly sensitive humidity response (57% RH) (Fig. 20h), Zhu et al. [97] demonstrated a new paper thin-film H_2_ sensor using Ti_3_C_2_T_x_ MXene nanosheets and palladium colloidal nanoclusters (Pb CNC) as activators. The MXene@Pd CNC paper film is easily prepared through a vacuum filtration process based on a fully colloidal solution (Fig. 21a). The paper film is flexible, lightweight, and has a dense, shiny surface. The obtained MXene@Pd CNC thin-film sensor exhibits moderate H_2_ response at room temperature in a flat or curved state (Fig. 21b). Specifically, MXene@Pd CNC thin-film sensor provides a response time of (32 ± 7)s and a sensitivity of S = (23.0 ± 4.0)% ± 4% H_2_ (Fig. 21c). In addition, the MXene@Pd CNC sensor can perform "in situ mode" H_2_ detection directly along a paper film of the required size. Intense H_2_ entrapment in the ultrafine palladium carbon nanotube lattice alters the work function and leads to MXene's electron codoping, explaining the underlying regime of gas induction (Fig. 21d). Muckleyet et al. [110] reported on ion intercalated MXenes (Ti_3_C_2_-K and Ti_3_C_2_-Mg) for humidity sensing (RT). Ion embedding increases the spacing between MXene layers and absorbs H_2_O molecules between the layers (Fig. 21e). The conclusion drawn from neutron scattering combined with theoretical calculations is that K^+^and Mg^2+^ ions cause each ion to embed 2 and 5 H_2_O molecules, respectively, indicating an increase in lattice parameters. They also found that the weight response of MXene to water is 10 times faster than their electrical response, indicating that the expansion/contraction of channels between MXene layers caused by H_2_O leads to the capture of H_2_O molecules as depletion charge dopants (Fig. 21f–i).

**Fig. 22:**
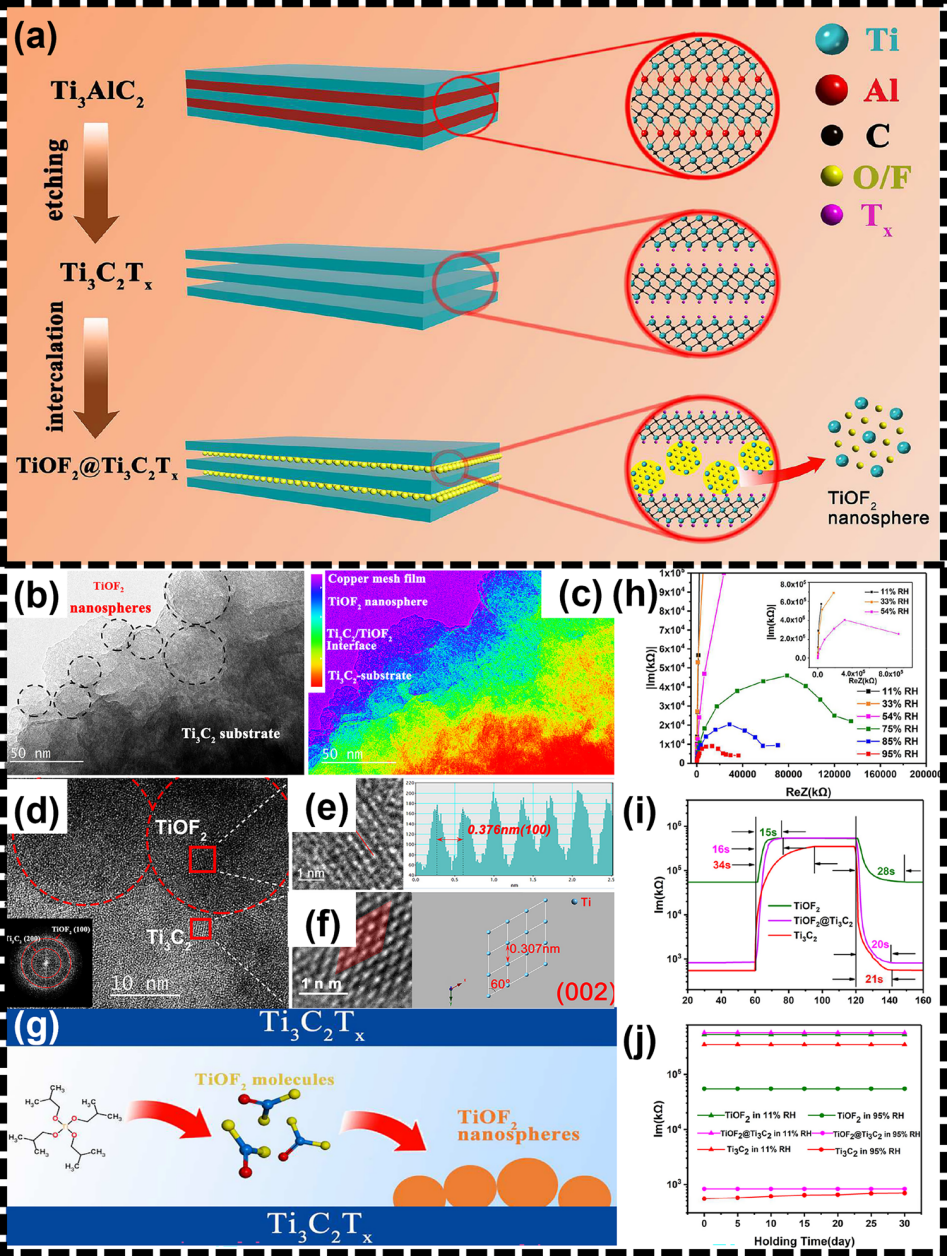
**a** Scheme for the fabrication of TiOF_2_@Ti_3_C_2_T_x_. **b** and** c** The cross section of the monolayer of the TiOF_2_@Ti_3_C_2_T_x_ sheet and the rainbow map to show the composition distribution in situ. **d** TEM image of TiOF_2_ nanospheres growing on the Ti_3_C_2_T_x_ substrate. **e** HRTEM image of TiOF_2_ nanospheres. **f** HRTEM image of Ti_3_C_2_ substrate. **g** Scheme for the hydrolysis and adsorption to synthesize TiOF_2_@Ti_3_C_2_T_x_. **h** Complex impedance property of TiOF_2_@Ti_3_C_2_T_x_ at the different RH. **i** Response and recovery properties of sensors with TiOF_2_, Ti_3_C_2_T_x_ and TiOF_2_@Ti_3_C_2_T_x_. **j** Three samples tested for extended stability at variable humidity. Reproduced with permission from Ref. [98]

Within other studies, the investigators tried to improve the sampling performance by doping iron molybdate (Fe_2_(MoO_4_)_3_) [107], Ni(OH)_2_ [106] and Ti_3_C_2_T_x_ MXene for H_2_ (in room temperature), n-butanol (in 120 ℃), and NH_3_ (in room temperature) sensing, respectively. In another study, transition metal fluoride oxide (TiOF_2_) was surface modified on Ti_3_C_2_T_x_ and subsequently used as a humidity sensor (Fig. 22a–f). By stabilizing the surface end groups, the MXene films showed improved reaction area, flexibility, and catalytic oxidation (Fig. 22j). In addition, the manufactured sensors exhibit good sensitivity and selectivity when exposed to humid environments [98] (Fig. 22h, i). Table 3 provides a detailed overview of sensors based on MXene nanocomposites. In conclusion, insertion of metallic ions and precious metals is also an effective way to improve the gas sensing performance of the original MXenes [109].

The new MXene gas sensor will be the next generation of universal sensors for future wearable electronic devices, with performance comparable to other 2D material sensors. Through Table 3, it can be clearly found that most of the reported 2D MXenes-based composites are suitable for sensing at room temperature. Secondly, MXenes-based composite materials have been tested for sensing different gases/VOCs and have been found to be highly sensitive to ammonia, acetone, ethanol, nitrogen dioxide, methane, and humidity. On the other hand, the application of MXene-based composites in gas sensors has advantages and disadvantages, as shown in Table 4.

**Table 4:** Advantages and disadvantages of MXene-based composite gas sensors

MXene-complex	Advantage	Shortcoming
MXene/rGO	The working temperature is room temperature, and the detection limit for various gases is low, with good sensitivity	The response recovery time at room temperature is relatively long, and the corresponding gas sensing mechanism of the composite material is unclear
MXene/metallic oxide	High sensitivity and high response to various VOC gases	The selectivity is poor, the working temperature cannot reach room temperature, and the stability is poor
MXene/TMDs	The reaction temperature is room temperature and has good stability	There is relatively little research, and the gas sensing response of composite materials is relatively low
MXene/MOF	It has high sensitivity in a dry environment and operates at room temperature	There is no gas sensitivity research on VOC gas
MXene/polymer	Composite materials are most suitable for use in humidity gas sensors and operate at room temperature	The minimum limit for detecting gas/VOC/humidity response is relatively high

## Gas Sensing Mechanism of MXenes

### MXenes Surface Adsorption Calculation

**Fig. 23:**
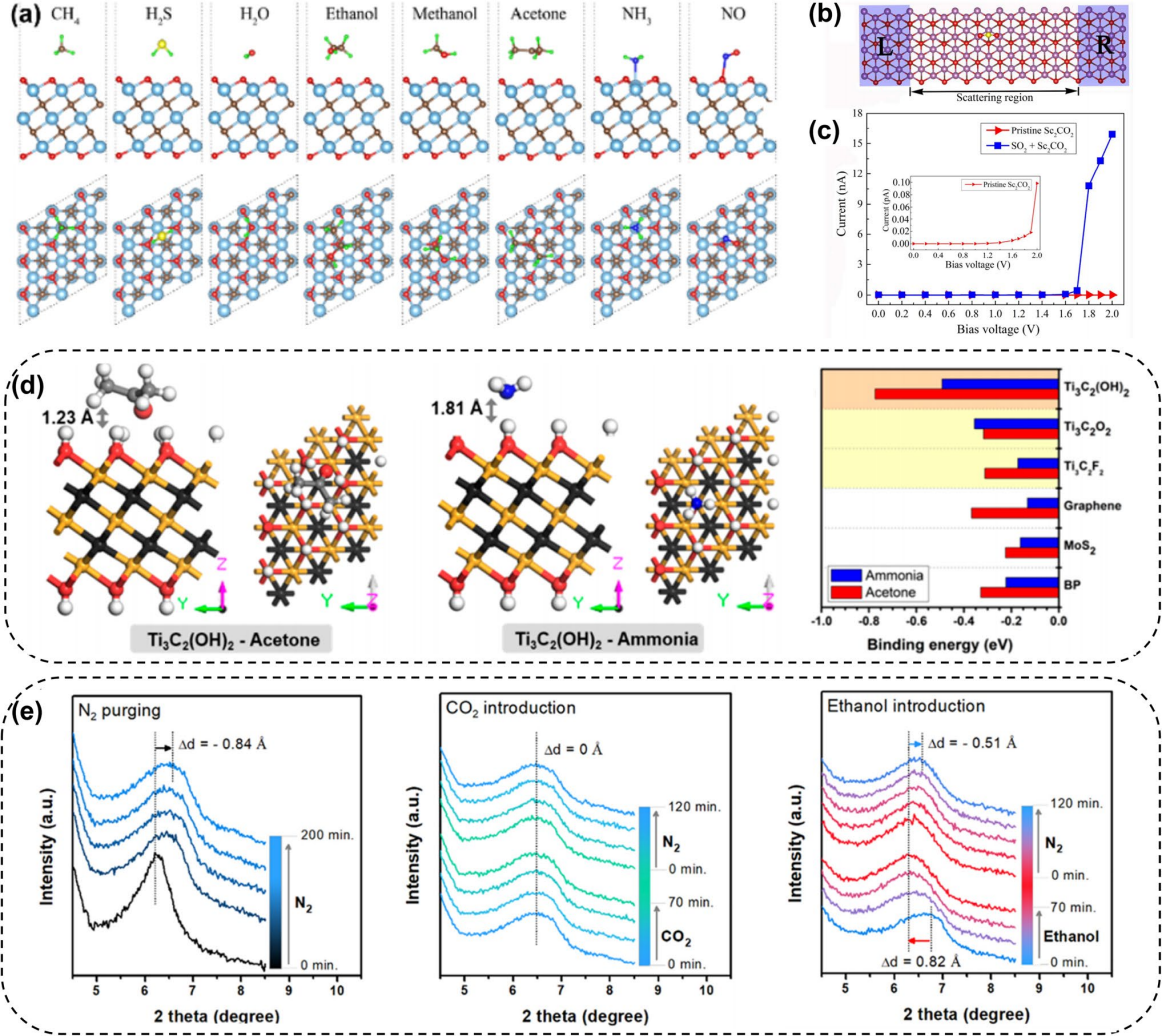
**a** Side and top views of the most stable configurations of different gas molecules adsorbed on the Ti_3_C_2_O_2_ surface. Reproduced with permission from Ref. [216]. **b** Two-probe model of monolayer Sc_2_CO_2_ sensor for detecting SO_2_ molecule. **c** Predicted I-V characteristics of Sc_2_ CO_2_ with SO_2_ molecules. Reproduced with permission from Ref. [218]. **d** Density functional theory (DFT) simulation results for gas molecules adsorbed on various 2D materials. Side and top views of the minimum energy configurations for acetone and ammonia on Ti_3_C_2_(OH)_2_. Minimum binding energies of acetone and ammonia on Ti_3_C_2_(OH)_2_, Ti_3_C_2_O_2_, Ti_3_C_2_F_2_, graphene, MoS_2_, and BP. Reproduced with permission from Ref. [42]. **e** The (002) peak shift of Ti_3_C_2_T_2_ film during N_2_ purging for 200 min. The (002) peak shift of Ti_3_C_2_T_x_ film during introduction of CO_2_ (1%) or ethanol (0.1%) for 70 min, followed by N_2_ purging for 120 min to purge out target gases. Reproduced with permission from Ref. [222].

It has been theoretically proven that MXenes with semiconductor properties (M_2_CO_2_, M = Sc, Ti, Zr, Hf) are highly sensitive to NH_3_, as shown in Fig. 23a. Xiao et al. [216] calculated and found that after NH_3_ was adsorbed as an electron donor on M_2_CO_2_, charge transfer mainly occurred between the M atom of M_2_CO_2_ and the N atom of NH_3_. When MXene adsorbed NH_3_, the charge of NH_3_ molecules was transferred to the transition metal atom on the surface of MXene, and the conductivity of Ti_2_CO_2_ was significantly improved. They also found that desorption of NH_3_ can be easily achieved by adjusting the electrons injected into M_2_CO_2_, making the NH_3_ sensor reversible [217]. For example, the lowest unoccupied electronic state (LUES) of Zr_2_CO_2_ mainly comes from Zr atoms, which means that when an additional electron is introduced into Zr_2_CO_2_, the electrons will fill the unoccupied electronic orbitals of Zr atoms. Therefore, the injected electrons are mainly distributed on the transition metal, leading to an increase in the metal bond length and adsorption energy of NH_3_-M, resulting in a decrease in the energy of NH_3_ adsorption on the MXene surface. The research team [218] also found that the single-molecule layer Sc_2_CO_2_ has good adsorption strength and obvious charge transfer for SO_2_. The transfer of charge from SO_2_ to Sc_2_CO_2_ increases the DOS at the Fermi level of Sc_2_CO_2_ and the conductivity of Sc_2_CO_2_. By applying external tensile strain or electric field, high selectivity, high sensitivity, controllable capture, or reversible desorption can be achieved, which predicts that Sc_2_CO_2_ has good sensing performance for toxic SO_2_ gas, as shown in Fig. 23b, c. The surface functional groups of MXenes have an undeniable contribution or impact on gas sensing performance. Junkaew et al. [219] used density functional theory (DFT) calculations to investigate the reactivity and selectivity of four O-functionalized MXenes, namely M_2_CO_2_ (M = Ti, V, Nb, Mo), toward gas molecules. According to the calculated adsorption energy results, among the 11 gas molecules, Ti_2_CO_2_ and Nb_2_CO_2_ have stronger adsorption capacity for NH_3_, while Mo_2_CO_2_ and V_2_CO_2_ are more sensitive to NO. The surface functional groups of Ti_3_C_2_T_x_ MXene material are a combination of -F, = O, and -OH. The presence and content changes of these functional groups can achieve selective sensing of gas molecules. For example, Pourfath et al. [220] studied through charge difference calculations that the contribution of surface functional groups to charge transfer is different. Fluorine atoms have a smaller contribution to charge transfer than oxygen atoms. Therefore, there is a strong electrostatic attraction between the lone pair electrons of the O atom in the = O functional group on MXenes and the positively charged part of the exposed hydrogen atom in NH_3_ molecules. Therefore, controlling the content of the = O functional group on the MXenes surface can improve the selectivity toward NH_3_ molecules. Recently, Naqvi et al. [221] explored several gases (such as CH_4_) through DFT calculations.

### First Principles Exploration of MXenes Gas-Sensitive Mechanisms

Maleski et al. [42] used DFT to simulate and calculate the binding energies of acetone and ammonia on Ti_3_C_2_T_x_, MoS_2_, RGO, and BP to study the sensing mechanism of Ti_3_C_2_T_x_ on acetone and NH_3_ gases, as shown in Fig. 23d. For the two gases of acetone and ammonia, Ti_3_C_2_(OH)_2_ exhibits the strongest binding energy more than twice that of other two-dimensional materials. It is speculated that the superior gas adsorption performance of hydroxyl groups in Ti_3_C_2_T_x_ is the main reason for its high sensitivity to acetone and ammonia. This work demonstrates the presence of charge transfer induced by gas adsorption in the gas sensing mechanism of MXenes. In addition, Zhou et al. [216] used Ti_3_C_2_T_x_ as a gas sensing material to test CH_4_, H_2_S, H_2_O, NH_3_, NO, ethanol, methanol, and acetone gases at room temperature, and found that Ti_3_C_2_T_x_ had very high selectivity for NH_3_. In order to understand the reason for this high selectivity, they also studied the adsorption behavior, adsorption energy, adsorption geometry, charge transfer, and other aspects using first principles calculation methods. They also confirmed that the charge transfer caused by NH_3_ adsorption on Ti_3_C_2_T_x_ is the main reason for the change in resistance of Ti_3_C_2_T_x_. However, MXenes have metal conductivity and contain interlayer water molecules, which means that gas molecules may interact in a more complex manner than typical charge transfer. Koh et al. [222] demonstrated the swelling effect of gas on Ti_3_C_2_T_x_ MXene materials by intercalating Ti_3_C_2_T_x_ with Na^+^ ions and using in situ XRD technology. After 70 min of ethanol blowing, the (002) peak of Ti_3_C_2_T_x_ shifted toward a smaller angle and the interlayer spacing increased by 0.82 Å. After 120 min of N_2_ blowing, the adsorbed ethanol was desorbed and the (002) peak of Ti_3_C_2_T_x_ recovered toward a larger angle. The interlayer spacing of Ti_3_C_2_T_x_ membrane decreased by 0.51 Å compared to that after ethanol swelling, as shown in Fig. 23e. Therefore, regulating the interlayer distance of Ti_3_C_2_T_x_ MXene is also very important for improving the selectivity of gas sensing.

## Summary and Outlook

Starting from the application of new MXene-based composites in the field of gas sensing, this article briefly introduces the preparation methods of gas sensing devices, the structure of MXene, and the properties related to gas sensing. It focuses on the research progress of MXene and graphene, metal oxides, TMDs, MOFs, and polymers in the field of gas sensing, and summarizes the gas sensing mechanism of MXene. However, the development of practical gas sensors based on MXene still faces many challenges:

1. It is necessary to develop green and safe macro preparation methods and surface functional group oriented regulation technologies for MXene. At present, the most mature preparation method for M_n+1_X_n_ is liquid-phase chemical etching, usually using ternary M_n+1_AX_n_ precursors as starting materials. In fluorinated solutions such as hydrofluoric acid (HF) and fluoride salts (LiF + HCl, NH_4_HF_2_), chemical etching selectively removes the A-layer elements in ternary M_n+1_AX_n_, achieving good selective etching effect and obtaining functional group-rich multilayer M_n+1_X_n_T_x_ materials. On the one hand, MAX phase is usually formed through high-temperature processing of titanium and aluminum, and requires several grinding processes to obtain fine MAX powder. On the other hand, using hydrofluoric acid or fluorinated salts as etching solvents, the highly toxic gases generated during the preparation process seriously endanger human and environmental safety. In addition, the etching capabilities of different solution systems vary, resulting in low two-dimensional yield and difficulty in optimizing the preparation process. This will result in high preparation costs for MXene materials and limit their large-scale application in the gas sensing field. More importantly, the fluorine containing solution reaction system inevitably leads to the random coexistence of three functional groups (= O, -F, -OH) on the surface of M_n+1_X_n_T_x_, making accurate control extremely difficult. The regulation of functional group states (types and quantities) by changing experimental conditions faces enormous challenges in experiments, and mature and feasible experimental methods for precise regulation of functional groups have not yet been formed, making it difficult to improve selectivity for specific gases through the design of surface functional groups.
2. The variety of MXene material systems still needs to be greatly expanded. Since the discovery of MXene materials in 2011, people's understanding of their structure is still in the initial stage, especially the lack of effective preparation techniques for the types of MXene materials predicted by theory. As a result, MXene currently used in the gas sensing field mainly focuses on two-dimensional Ti_3_C_2_T_x_ and its composite materials. For the large number of MXene material families, more innovative preparation methods have been developed to synthesize pure MXene materials with more diverse types. The combination of surface modification, element doping, heterogeneous recombination and other means to design the composition of the material is a technical bottleneck in expanding the application of MXene in the gas sensing field.
3. The interaction mechanism between MXene and gas molecules needs to be further studied. Due to the richer atomic species and combination types of MXene compared to traditional two-dimensional materials such as graphene, the surface adsorption and charge transfer mechanisms in gas-sensitive processes will be more complex. Whether it is oxidizing or reducing, it is observed that all adsorbed gas molecules will cause an increase or decrease in resistance with a high signal-to-noise ratio. At the same time, interlayer expansion also has a significant impact on the conductivity changes and gas response of the material.

At present, research on MXene is still in its infancy, providing a basic building block for gas-sensitive materials. Experimental data and computational predictions indicate that by selecting over 60 sets of available layered ternary carbides and nitrides, stable structures of different types of MXene can be obtained. It is expected that MXene and its composites will have unlimited potential in the field of gas sensing.
